# Synthesis of a novel category of pseudo-peptides using an Ugi three-component reaction of levulinic acid as bifunctional substrate, amines, and amino acid-based isocyanides

**DOI:** 10.3762/bjoc.15.82

**Published:** 2019-04-04

**Authors:** Maryam Khalesi, Azim Ziyaei Halimehjani, Jürgen Martens

**Affiliations:** 1 Faculty of Chemistry, Kharazmi University, P.O. Box 15719-14911, 49 Mofateh Street, Tehran, Iran; 2 Institut für Chemie (IfC), Carl von Ossietzky Universität Oldenburg, P.O. Box 2503, 26111 Oldenburg, Germany

**Keywords:** amino acid-based isocyanides, levulinic acid, multicomponent, pseudo-peptides, Ugi reaction

## Abstract

The synthesis of a novel category of pseudo-peptides via intramolecular Ugi reaction of levulinic acid (4-oxopentanoic acid), aromatic and aliphatic amines, and amino acid-based isocyanides is reported. Levulinic acid was applied as a bifunctional substrate containing both carbonyl and acid moieties suitable for the Ugi reaction. This article provides a facile and convenient one-pot procedure for the synthesis of peptide-like heterocyclic molecules containing 2-pyrrolidone (γ-lactam), amide and ester functional groups with good to excellent yields.

## Introduction

The multistep synthesis of complex molecules normally requires a large number of repetitive synthetic operations, such as extraction, separation, chromatography and other purification steps. These disadvantages encouraged chemists to synthesize complex molecules using multicomponent reactions (MCRs). MCRs transform three or more starting materials into a single product in an atom- and step-economical way in diversity- and target-oriented syntheses in modern organic synthesis. In addition, MCRs are characterized by high yields, time efficiency, low waste production, and reduced energy consumption [1–9]. So, the design of novel MCRs with facile and green processes has fascinated considerable attention in the fields of drug discovery, organic synthesis of natural products, and materials sciences [10].

Undoubtedly, one of the most prominent and studied MCRs is the Ugi reaction. The Ugi four-component condensation reaction (U-4CC) between an aldehyde, an amine, a carboxylic acid and an isocyanide provides a rapid preparation of α-aminoacyl amide or pseudo-peptide derivatives. These biologically active peptide-like molecules can be utilized to circumvent some of the problems associated with several natural peptides such as stability against proteolysis, poor bioavailability, receptor selectivity, and short duration of action [11]. The Ugi reaction allows the introduction of several substituents in its adducts to prepare novel peptidomimetics with potential pharmaceutical applications. Therefore, the development of innovative Ugi reactions is crucial for the synthesis of novel chemical libraries for various purposes [12].

In recent years, one of the modifications for Ugi reactions is the introduction of bifunctional substrates into the Ugi condensation reaction in order to keep the multicomponent sequence as short as possible which makes it less complicated [13–17].

Levulinic acid or 4-oxopentanoic acid, is an organic compound which is classified as a ketoacid. It can be easily prepared in industrial scale and low price by acid catalysis from renewable resources, such as sugars, lignocellulosic biomass and waste materials [18]. It can be used as a bifunctional precursor for the synthesis of pharmaceuticals, plasticizers, and different additives [19]. Furthermore, it is recognized as an excellent starting material for Ugi reactions because it has two functional groups in its structure. By the way, using bifunctional chemicals in Ugi four-component condensation reaction (4CC) converts it to an Ugi three-component condensation reaction (3CC) and this is identified as an Ugi-4-centre-3-component reaction (U-4C-3CR) [20–23]. This reaction proceeds through an intramolecular mechanism which leads to the formation of heterocyclic products as a result of a ring-closure process.

In 1998, Ugi et al. reported the intramolecular Ugi-4C-3CR of ketoacids such as levulinic acid and phthalaldehydic acid with aliphatic amines and ordinary isocyanides with excellent yields [24]. In 2003, Mironov et al. reported the Ugi reaction of levulinic acid, isocyanides and primary amines in aqueous media with high yields [25]. In addition, Banfi et al. have shown that by using levulinic acid in multicomponent reactions and post-multicomponent reactions, diversities of bicyclic drug-like heterocyclic compounds can be obtained [26].

## Results and Discussion

**Scheme 1:**
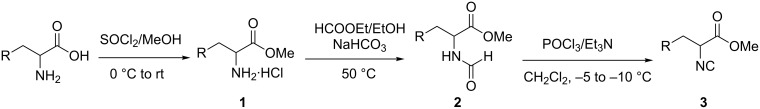
Synthesis of amino acid-based isocyanides starting from α-amino acids.

In continuation of our interest on the synthesis of novel pseudo-peptides [27–29] via multicomponent reactions, herein we investigate the Ugi-4C-3CR of levulinic acid, aromatic and aliphatic amines and amino acid-based isocyanides. First of all, racemic α-amino acids such as DL-tryptophan, DL-phenylalanine and DL-leucine were used as amine source for the synthesis of isocyanide esters **3** through three sequential reactions [30–31]. The first reaction is esterification of the α-amino acid using thionyl chloride in methanol as reagent and solvent. The second reaction is the formylation of the corresponding amino acid ester salt with ethyl formate in the presence of NaHCO_3_. Finally, the formamide group was transformed to the corresponding isocyanide **3** using POCl_3_ and triethylamine (Scheme 1).

**Scheme 2:**
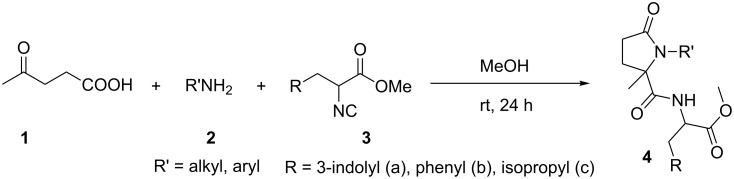
Synthesis of pseudo-peptides using levulinic acid, isocyanide esters and amines.

**Figure 1:**
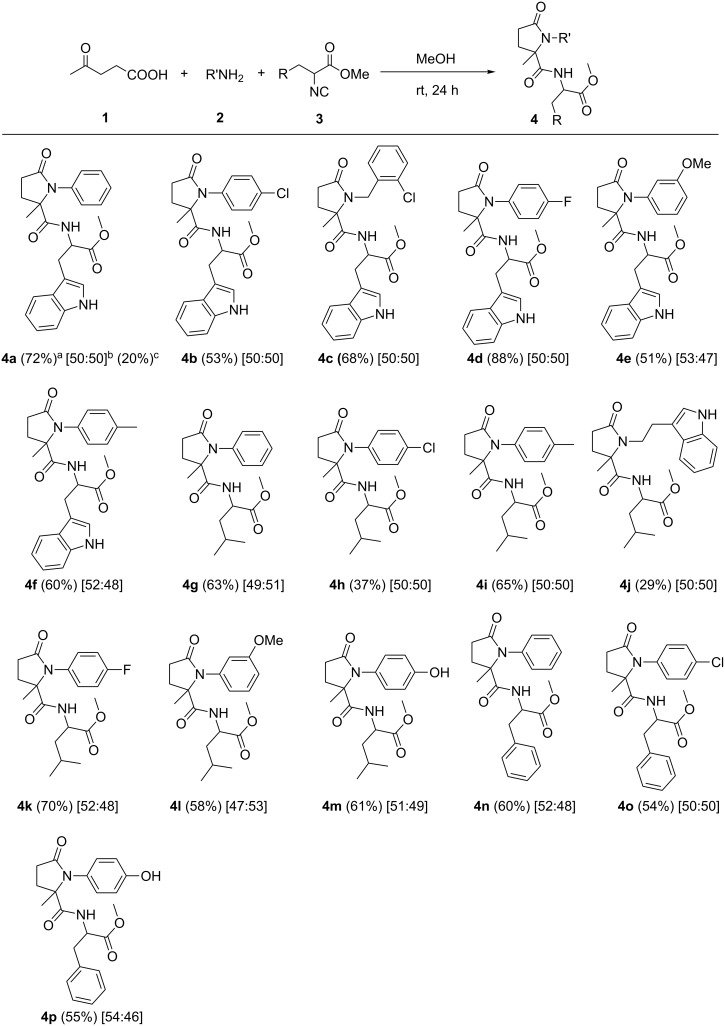
Synthesis of functionalized 5-membered lactams using Ugi reaction. ^a^Isolated yield for mixture of diastereomers. ^b^The ratio of diastereomers was determined by ^1^H NMR analysis. ^c^Isolated yield of one of the diastereomers as pure stereoisomer after several recrystallization steps.

The prepared amino acid-based isocyanides **3** were applied in an Ugi-4C-3CR (Scheme 2). For this purpose, the reactions of one equivalent of bifunctional substrate levulinic acid (**1**), an amine **2** and an isocyanide ester **3** were carried out in methanol as solvent at room temperature to produce the corresponding pseudo-peptides **4**. We observed that under the optimized reaction conditions, good to high yields of products were obtained. The results are summarized in Figure 1. Various aromatic and aliphatic amines were applied in this protocol to give the corresponding pseudo-peptides. For this purpose, aliphatic amines such as *o*-chlorobenzylamine and tryptamine and aromatic amines such as aniline, 4-chloroaniline, 4-aminophenol, *p*-toluidine, 4-fluoroaniline, and *m*-anisidine were applied successfully in this protocol. In addition, all three prepared isocyanides from DL-tryptophane, DL-leucine and DL-phenylalanine worked very well in this reaction to provide the corresponding pseudo-peptides **4** containing γ-lactam, amide and ester functional groups in a single structure.

By starting from DL-amino acids, the corresponding racemic isocyanides were obtained. By using the racemic isocyanides in the Ugi-4C-3CR, a mixture of diastereomers were obtained in approximately 1:1 ratio (see NMR spectra in Supporting Information File 1). Attempts to find a suitable procedure for separation of the diastereomers without losing the yield was not successful. In the case of **4a**, one of the diastereomers [(R*,S*)-**4a**] was obtained as a pure compound in low yield (20%) after several recrystallization steps from MeOH.

**Scheme 3:**
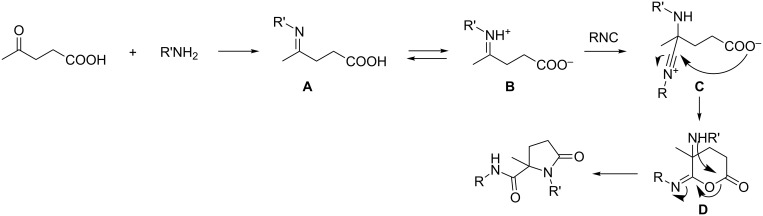
Proposed mechanism for Ugi-4C-3CR.

A proposed mechanism for this reaction is depicted in Scheme 3. The primary step in the mechanism is the condensation reaction of the carbonyl group of levulinic acid with an amine component that leads to the formation of an imine intermediate **A**. The formed Schiff base is in equilibrium with its iminium cation **B** as a result of an intramolecular proton exchange with the carboxylic acid moiety which activates the iminium ion for the nucleophilic addition of isocyanide. Consequently, the electrophilic centre of the iminium ion in **B** is subjected to a nucleophilic attack of the isocyanide to furnish the intermediate **C**. Then, a second nucleophilic addition takes place at this nitrilium intermediate **C** with an intramolecular nucleophilic addition of the carboxylate anion. The final step is an acyl transfer from oxygen to nitrogen (Mumm rearrangement) in **D** which completes the Ugi reaction accompanied by formation of the corresponding bis-amides.

**Figure 2:**
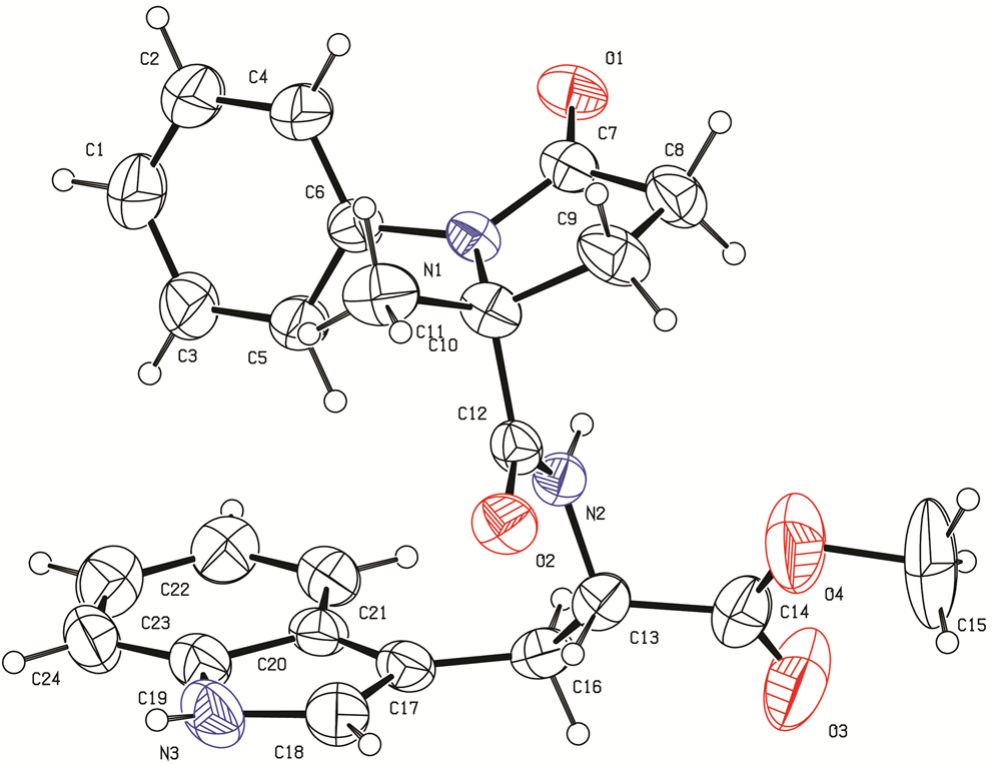
ORTEP representation of compound (R*,S*)-**4a** with thermal ellipsoids at 50% probability. Opposite enantiomer is omitted for clarity. The atom numbering does not follow IUPAC nomenclature.

The structure of products was confirmed by IR, ^1^H and ^13^C NMR, CHN and HRMS analyses and by X-ray crystallography for **4a**. The IR spectra of the derivatives show characteristic absorbance bands at 3200–3500 cm^−1^ for the N–H bond stretching vibration and at 1640–1750 cm^−1^ for two carbonyls of the amide groups and one carbonyl of the ester group. The ^1^H NMR spectra of the products show a characteristic peak at 6–7 ppm for the amide hydrogen and a peak as multiplet at 4.40–5.00 ppm for the CH group in the stereogenic center. Carbons of the amide and ester moieties were observed around 170–177 ppm in ^13^C NMR spectra. In addition, the structure of compound (R*,S*)-**4a** was conﬁrmed by single crystal X-ray diffraction and an ORTEP representation is shown in Figure 2 (CCDC 1896942); for details of the crystal structure data and refinement of (R*,S*)-**4a** see Supporting Information File 1).

## Conclusion

In conclusion, we have synthesized a novel category of pseudo-peptides containing γ-lactam, amide and ester moieties in a single structure via Ugi-4-centre-3-component reaction. The main advantage of this paper refers to the application of three amino acid-based isocyanides in the reaction with levulinic acid as a bifunctional substrate and amines which led to the formation of novel peptidomimetics with potential biological activities. In addition, the presence of an ester functional group in the structure of products makes them suitable substrates for further derivatization.

## Supporting Information

File 1 Experimental procedures, characterization data and copies of ^1^H and ^13^C NMR spectra of all compounds.
