# Supplementary material for: Synthesis of a novel category of pseudo-peptides using an Ugi three-component reaction of levulinic acid as bifunctional substrate, amines, and amino acid-based isocyanides
Source: Beilstein J Org Chem. 2019 Apr 4;15:852–7. doi: 10.3762/bjoc.15.82 (PMC6466677; doi:10.3762/bjoc.15.82)
Supplement: File 1 — Experimental procedures, characterization data and copies of 1H and 13C NMR spectra of all compounds. [file Beilstein_J_Org_Chem-15-852-s001.pdf]

BEILSTEIN JOURNAL OF ORGANIC CHEMISTRY

Supporting Information

for

Synthesis of a novel category of pseudo-peptides using an Ugi three-component reaction of levulinic acid as bifunctional substrate, amines, and amino acid-based isocyanides

Maryam Khalesi, Azim Ziyaei Halimehjani and Jürgen Martens

Beilstein J. Org. Chem. **2019,** *15,* 852–857. doi:10.3762/bjoc.15.82

Experimental procedures, characterization data and copies of ^{1}H and ^{13}C NMR spectra of all compounds

License and Terms: This is a supporting information file under the terms of the Creative Commons Attribution License (http://creativecommons.org/licenses/by/4.0). Please note that the reuse, redistribution and reproduction in particular requires that the authors and source are credited.
The license is subject to the *Beilstein Journal of Organic Chemistry* terms and conditions: (https://www.beilstein-journals.org/bjoc)

**Content**	**Pages**
General experimental procedure and characterization data for all compounds	S2–S6
Copies of ^{1}H and ^{13}C NMR spectra for all compounds	S7–S22
Crystal data of compound (*R**,*S**)-**4a**	S23

General experimental procedure and characterization data for all compounds

General. All reagents and solvents were analytically pure and were used as received. Column chromatographic purification was carried out using SiO_2 (0.040-0.060 mm, type KG 60). TLC was performed on Macherey-Nagel SiO_2 F254 plates on aluminum sheets. Melting points were determined with a Branstead Electrothermal 9200 apparatus and are uncorrected. IR spectra were recorded on a Perkin-Elmer FT RX1spectrophotometer over the range 400–4000 cm^{-1}. ^{1}H and ^{13}C NMR spectra of isolated products were recorded on a Bruker AMX R500 (measuring frequency: ^{1}H NMR = 500.1 MHz, ^{13}C NMR = 125.8 MHz) or a Bruker Avance III 500 (measuring frequency: ^{1}H NMR = 499.9 MHz, ^{13}C NMR = 125.7 MHz) in $CDCl_3$ solution. HRMS (high-resolution mass spectra) were measured on a THERMO SCIENTIFIC Advantage and a THERMO SCIENTIFIC Exactive instrument. Elemental analyses were conducted with a Perkin-Elemer 2004 (II) CHN analyzer.

General procedure for the synthesis of amino acid-based isocyanides 3a–c

(a) General procedure for the preparation of DL-amino acid methyl ester hydrochlorides: DL-amino acid (50 mmol) was dissolved in MeOH (550 mL) and the solution was cooled in an ice/salt bath. Then, thionyl chloride (50 mmol) was added dropwise for 1 hour. The reaction temperature was maintained between −5–0 °C during the addition. Then, the reaction mixture was stirred for 24 h at room temperature. Finally, the reaction mixture was concentrated on a rotary evaporator to give the crude product. The crude product was washed with DCM (two times) to give the corresponding pure racemic amino acid ester hydrochloride.

(b) General procedure for the formylation of DL-amino acid methyl ester hydrochlorides: *DL*-aminoacid methyl ester hydrochloride (20 mmol) was dissolved in ethyl formate (100 mL)/EtOH (30 mL) mixture. Then, sodium bicarbonate (20 mmol) was added to the flask. The reaction mixture was stirred at 50 °C for 2 days. After the completion of the reaction, the mixture was filtered for removing the excess amount of sodium bicarbonate. Then, the filtrate was concentrated on a rotary evaporator to give the product.

(c) General procedure for the transformation of DL-formamide to DL-isocyanide: *DL*-formylated amino acid methyl ester (20 mmol) was dissolved in DCM (200 mL) and TEA (100 mmol) was added to the solution at room temperature. Then the reaction mixture was cooled in an ice/salt bath to maintain the temperature between −10 and −5 °C. Then, phosphorus oxychloride (20 mmol) was added dropwise to the reaction mixture and was stirred for 2 h. The reaction mixture was quenched by aqueous $NaHCO_3$ and the organic phase was separated, dried over Na_2SO_4, and evaporated under reduced pressure to give a precipitate. The precipitate was purified by column chromatography using DCM as solvent.

General procedure for the synthesis of Ugi adducts:

A solution of levulinic acid (**1**, 1 mmol) and an amine **2** (1 mmol) in MeOH (5 mL) was stirred for 0.5 h at room temperature. Then, *DL*-amino acid–based isocyanide **3** (1 mmol) was added to the reaction mixture at the same temperature. The reaction progress was monitored by TLC. After 24 h, the solvent was removed under reduced pressure and the precipitate purified by column chromatography using petroleum ether and ethyl acetate mixture as eluent (SiO_2, PE/EtOAc; 2:1 v/v). All products were obtained as 1:1 mixture of diastereomers after column chromatography as determined by ^{1}H NMR spectroscopy. All compounds were characterized using IR, ^{1}H NMR, ^{13}C NMR and HRMS or CHN analyses.

Methyl (2-methyl-5-oxo-1-phenylpyrrolidine-2-carbonyl)tryptophanate (**4a**): White crystalline solid (yield, 318 mg, 72%); mp: 158 – 159 °C; ^{1}H NMR (500 MHz, Chloroform-*d*) δ 1.35 (s, 3H), 2.00 (m, 1H), 2.42 – 2.54 (m, 2H), 2.72 (m, 1H), 3.26 (dd, *J* = 14.9 and 7.9 Hz, 1H), 3.41 (m, 1H), 3.77 (s, 3H), 4.90 (m, 1H), 6.52 (d, *J* = 7.1 Hz, 1H), 6.84 (d, *J* = 2.5 Hz, 1H), 6.91 – 6.94 (m, 2H), 7.08 – 7.18 (m, 4H), 7.21 – 7.25 (m, 1H), 7.32 (m, 1H), 7.55 (dd, *J* = 8.0 and 1.0 Hz, 1H), 8.13 (brs, 1H) ppm; ^{13}C NMR (126 MHz, Chloroform-*d*) δ 22.3, 27.2, 30.3, 34.1, 52.4, 52.5, 68.3, 109.4, 111.5, 118.3, 119.8, 122.6, 122.7, 122.8, 125.6, 126.9, 128.9, 135.9, 136.4, 172.0, 173.4, 175.4 ppm; IR (KBr): *ν* 3328, 1736, 1690, 1659, 1595, 1381, 1229, 748 cm^{-1}; Anal. calcd for $C_{24}H_{25}N_3O_4$ (419.47) C, 68.72; H, 6.01; N, 10.02; Found: C, 68.42; H, 6.04; N, 9.88%.

The (*R**,*S**)-diastereomer (*R**,*S**)-**4a** was obtained from **4a** (a mixture of two racemic diastereomers) as a pure compound after several recrystallization steps from MeOH.

Methyl (1-(4-chlorophenyl)-2-methyl-5-oxopyrrolidine-2-carbonyl)tryptophanate (**4b**): White crystalline solid (yield, 240 mg, 53%); mp: 155 – 156 °C; ^{1}H NMR (500 MHz, Chloroform-*d*) δ 1.33 (s, 3H), 1.89 (m, 1H), 2.13 (m, 1H), 2.31 (m, 1H), 2.51 (m, 1H), 3.20 (m, 1H), 2.37 (m, 1H), 3.80 (s, 3H), 4.91 (dd, *J* = 7.3 and 5.3 Hz, 1H), 6.79 – 6.85 (m, 2H), 6.86 (s, 1H), 6.96 – 7.04 (m, 2H), 7.14 (m, 1H), 7.27 (m, 1H), 7.36 (t, *J* = 8.0 Hz, 2H), 7.54 (d, *J* = 7.9 Hz, 1H), 8.11 (brs, 1H) ppm; ^{13}C NMR (126 MHz, Chloroform-*d*) δ 22.6, 27.1, 29.7, 30.2, 33.8, 52.5, 68.4, 109.4, 111.5, 118.3, 122.6, 122.8, 126.6, 126.9, 127.6, 129.1, 132.6, 134.4, 136.3, 172.05, 172.9, 175.6 ppm; IR (KBr): *ν* 3259, 1746, 1691, 1644, 1358, 1221, 822, 739 cm^{-1}; HRMS calcd for $C_{24}H_{24}ClN_3O_4$ $[M + Na]^+$ 476.1353; Found . 476.1351.

Methyl (1-(2-chlorobenzyl)-2-methyl-5-oxopyrrolidine-2-carbonyl)tryptophanate (**4c**): Yellow crystalline solid (yield, 320 mg, 68%); mp 150 – 154 °C; ^{1}H NMR (500 MHz, Chloroform-*d*) δ 1.28 (s, 3H), 1.86 (m, 1H), 2.17 (m, 1H), 2.32 (m, 1H), 2.47 (m, 1H), 3.18 (m, 1H), 3.31 (m, 1H), 3.74 (s, 3H), 4.03 (d, *J* = 16.3 Hz, 1H), 4.62 (d, *J* = 16.4 Hz, 1H), 4.79 (m, 1H), 6.99 (d, *J* = 2.3 Hz, 1H), 7.10–7.45 (m, 8H), 7.53 (m, 1H), 8.34 (brs, 1H) ppm; ^{13}C NMR (126 MHz, Chloroform-*d*) δ 22.4, 27.1, 29.3, 32.9, 41.4, 41.7, 52.6, 67.5, 109.5, 111.5, 118.3, 119.8, 122.5, 127.0, 127.1, 128.2, 128.5, 128.8, 129.3, 129.4, 134.7, 136.2, 171.8, 173.0, 176.3

ppm; IR (KBr): *ν* 3261, 1749, 1683, 1656, 1539, 1202, 740 cm^{-1}; Anal. calcd for $C_{25}H_{26}ClN_3O_4$ (467.1612) C, 64.17; H, 5.60; N, 8.98; Found: C, 64.12; H, 5.72; N, 8.58%.

Methyl (1-(4-fluorophenyl)-2-methyl-5-oxopyrrolidine-2-carbonyl)tryptophanate (**4d**): Cream solid (yield, 385 mg, 88%); mp: 114 – 115 °C; ^{1}H NMR (500 MHz, Chloroform-*d*) *δ* 1.31 (s, 3H), 1.96 (m, 1H), 2.34 (m, 1H), 2.45 (m, 1H), 2.74 (m, 1H), 3.24 (m, 1H), 3.40 (m, 1H), 3.81 (s, 3H), 4.89 (m, 1H), 6.45 (d, *J* = 6.2 Hz, 1H), 6.75 – 7.24 (m, 7H), 7.35 (m, 1H), 7.55 (dd, *J* = 7.9 and 1.0 Hz, 1H), 8.16 (brs, 1H) ppm; ^{13}C NMR (126 MHz, Chloroform-*d*) *δ* 22.5, 27.2, 29.8, 33.9, 52.5, 52.6, 68.3, 109.5, 111.4, 115.9 (d, $^2J_{C\text{-}F}$ = 25 Hz), 118.3, 119.9, 122.6, 126.9, 127.7, 128.5, 131.6, 136.3, 160.3 (d, $^1J_{C\text{-}F}$ = 246 Hz), 172.0, 173.0, 175.6 ppm; IR (KBr): *ν* 3302, 1743, 1694, 1657, 1372, 1218, 839, 744 cm^{-1}; HRMS calcd for $C_{24}H_{24}FN_3O_4$ $[M + Na]^+$ 460.1649; Found: 460.1643.

Methyl (1-(3-methoxyphenyl)-2-methyl-5-oxopyrrolidine-2-carbonyl)tryptophanate (**4e**): Viscous oil (yield, 224 mg, 51%); ^{1}H NMR (500 MHz, Chloroform-*d*) *δ* 1.38 (s, 3H), 1.96 (m, 1H), 2.17 (m, 1H), 2.42 (m, 1H), 2.70 (m, 1H), 3.28 (m, 1H), 3.37 (m, 1H), 3.68 (s, 3H), 3.76 (s, 3H), 4.87 (m, 1H), 6.46 (m, 1H), 6.54 (m, 1H), 6.70 – 7.05 (m, 3H), 7.11 (m, 1H), 7.17 – 7.25 (m, 2H), 7.33 (dd, *J* = 7.9 and 1.0 Hz, 1H), 7.53 (dd, *J* = 8.0 and 1.0 Hz, 1H), 8.18 (brs, 1H) ppm; ^{13}C NMR (126 MHz, Chloroform-*d*) *δ* 22.4, 22.6, 27.2, 29.9, 34.0, 52.4, 55.25, 68.6, 109.4, 111.5, 111.8, 112.4, 118.4, 118.9, 119.8, 122.5, 122.8, 122.9, 129.6, 136.3, 137.0, 159.9, 172.0, 173.2, 175.5 ppm; IR (KBr): *ν* 3327, 1741, 1658, 1602, 1363, 1211, 745 cm^{-1}; HRMS calcd for $C_{25}H_{27}N_3O_5$ $[M + Na]^+$ 472.1848; Found. 472.1840.

Methyl (2-methyl-5-oxo-1-(p-tolyl)pyrrolidine-2-carbonyl)tryptophanate (**4f**): Cream viscous oil (yield, 260 mg, 60%); ^{1}H NMR (500 MHz, Chloroform-*d*) *δ* 1.34 (s, 3H), 1.86 – 2.20 (m, 2H), 2.29 (s, 3H), 2.48 (m, 1H), 2.70 (m, 1H), 3.28 (m, 1H), 3.38 (m, 1H), 3.77 (s, 3H), 4.88 (m, 1H), 6.52 (brs, 1H), 6.79 – 6.93 (m, 3H), 7.07 – 7.15 (m, 3H), 7.24 (m, 1H), 7.34 (d, *J* = 8.1 Hz, 1H), 7.44 (d, *J* = 8.0 Hz, 1H), 8.18 (brs, 1H) ppm; ^{13}C NMR (75 MHz, Chloroform-*d*) *δ* 20.7, 22.2, 26.9, 27.4, 29.6, 33.7, 52.1, 68.2, 109.1, 111.2, 118.2, 119.5, 122.2, 122.6, 125.4, 126.6, 129.3, 132.8, 136.1, 136.5, 171.82, 173.2, 175.4 ppm; IR (KBr): *ν* 3270, 1734, 1692, 1648, 1513, 1374, 1201, 819, 741 cm^{-1}; HRMS calcd for $C_{25}H_{27}N_3O_4$ $[M + Na]^+$ 456.1899; Found: 456.1894.

Methyl (2-methyl-5-oxo-1-phenylpyrrolidine-2-carbonyl)leucinate (**4g**): Cream solid (yield, 218 mg, 63%); mp: 107 – 108 °C; ^{1}H NMR (500 MHz, Chloroform-*d*) *δ* 0.92 – 0.97 (m, 6H), 1.50 (s, 3H), 1.54 (m, 1H), 1.69 (m, 1H), 2.10 (m, 1H), 2.52 (m, 1H), 2.56 – 2.76 (m, 2H), 2.88 (m, 1H), 3.76 (s, 3H), 4.65 (m, 1H), 6.37 (d, *J* = 8.2 Hz, 1H), 7.25 – 7.45 (m, 5H) ppm; ^{13}C NMR (126 MHz, Chloroform-*d*) *δ* 21.45, 22.8, 23.0, 25.0, 30.3, 34.3, 40.8, 51.1, 52.4, 68.6, 125.4, 127.1, 129.0, 136.0, 173.0, 173.6, 175.5 ppm; IR (KBr): *ν* 3308, 1755, 1739, 1694, 1597, 1534, 1381, 1222, 1020, 764, 697 cm^{-1}; Anal. calcd for $C_{19}H_{26}N_2O_4$ (346.1893) C, 65.87; H, 7.56; N, 8.09; Found: C, 65.52; H, 8.0; N, 7.71%.

Methyl (1-(4-chlorophenyl)-2-methyl-5-oxopyrrolidine-2-carbonyl)leucinate (**4h**): Creamy viscous oil (yield, 140 mg, 37%); ^{1}H NMR (500 MHz, Chloroform-*d*) δ 0.91 – 0.94 (m, 6H), 1.48 (s, 3H), 1.61 (m, 1H), 1.67 (m, 1H), 2.10 (m, 1H), 2.47 (m, 1H), 2.61 (m, 1H), 2.67 (m, 1H), 2.73 (m, 1H), 3.75 (s, 3H), 4.63 (m, 1H), 6.69 (d, J = 8.4 Hz, 1H), 7.24 –7.39 (m, 4H) ppm; ^{13}C NMR (126 MHz, Chloroform-*d*) δ 21.4, 22.7, 25.0, 27.5, 30.30, 34.1, 40.5, 51.1, 52.4, 68.4, 126.7, 129.1, 132.3, 134.6, 173.0, 173.3, 175.6 ppm; IR (KBr): ν 3327, 1743, 1700, 1671, 1526, 1495, 1367, 1199, 828 cm^{-1}; HRMS calcd for $C_{19}H_{25}ClN_2O_4$ [M + Na]$^+$ 403.1401; Found: 403.1395.

Methyl (2-methyl-5-oxo-1-(p-tolyl)pyrrolidine-2-carbonyl)leucinate (**4i**): Cream solid (yield, 234 mg, 65%); mp: 108 – 110 °C; ^{1}H NMR (500 MHz, Chloroform-*d*) δ 0.90 – 0.98 (m, 6H), 1.48 (s, 3H), 1.57 (m, 1H), 1.69 (m, 1H), 2.09 (m, 1H), 2.36 (s, 3H), 2.48 – 2.61 (m, 2H), 2.69 (m, 1H), 2.87 (m, 1H), 3.75 (s, 3H), 4.63 (m, 1H), 6.49 (d, J = 8.2 Hz, 1H), 7.16 – 7.30 (m, 4H) ppm; ^{13}C NMR (126 MHz, Chloroform-*d*) δ 21.0, 21.5, 22.8, 22.9, 24.9, 30.3, 34.3, 40.7, 51.1, 52.3, 68.5, 126.4, 129.7, 133.5, 137.1, 173.05, 173.7, 175.7 ppm; IR (KBr): ν 3289, 1748, 1674, 1609, 1537, 1387, 1221, 817 cm^{-1}; HRMS calcd for $C_{20}H_{28}N_2O_4$ [M + Na]$^+$ 383.1947; Found: 383.1942 .

Methyl (1-(2-(1H-indol-3-yl)ethyl)-2-methyl-5-oxopyrrolidine-2-carbonyl)leucinate (**4j**): Creamy viscous oil (yield, 120 mg, 29%); ^{1}H NMR (500 MHz, Chloroform-*d*) δ 0.89 (d, J = 6.1 Hz, 6H), 1.23 – 1.34 (m, 2H), 1.56 (s, 3H), 1.98 (m, 1H), 2.37 (m, 1H), 2.46 – 2.51 (m, 2H), 3.06 (m, 1H), 3.13–3.23 (m, 2H), 3.39 (m, 1H), 3.67 (s, 3H), 3.79 (m, 1H), 4.47 (m, 1H), 6.08 (d, J = 8.0 Hz, 1H), 7.12 – 7.24 (m, 3H), 7.38 (m, 1H), 7.69 (d, J = 7.9 Hz, 1H), 8.03 (brs, 1H); ^{13}C NMR (75 MHz, Chloroform-*d*) δ 21.5, 22.6, 22.7, 24.5, 24.9, 29.6, 33.2, 40.3, 42.3, 50.9, 52.2, 67.6, 111.1, 112.7, 118.7, 119.3, 121.9, 122.08, 127.2, 136.2, 173.0, 174.1, 176.3 ppm; IR (KBr): ν 3297, 1743, 1672, 1654, 1555, 1407, 1339, 1227, 760 cm^{-1}; HRMS calcd for $C_{23}H_{31}N_3O_4$ [M + Na]$^+$ 436.2212; Found . 436.2207; Anal. calcd for $C_{23}H_{31}N_3O_4$ (413.2315) C, 66.81; H, 7.56; N, 10.16; Found: C, 67.05; H, 7.63; N, 9.75%.

Methyl (1-(4-fluorophenyl)-2-methyl-5-oxopyrrolidine-2-carbonyl)leucinate (**4k**): Viscous oil (yield, 255 mg, 70%); ^{1}H NMR (500 MHz, Chloroform-*d*) δ 0.90 – 0.96 (m, 6H), 1.47 (s, 3H), 1.53 – 1.63 (m, 2H), 1.65 – 1.71 (m, 1H), 2.08 (m, 1H), 2.46 – 2.59 (m, 2H), 2.65 – 2.76 (m, 1H), 3.75 (s, 3H), 4.63 (m, 1H), 6.56 (d, J = 8.2 Hz, 1H), 7.04 – 7.12 (m, 2H), 7.26 (m, 1H), 7.38 (m, 1H) ppm; ^{13}C NMR (126 MHz, Chloroform-*d*) δ 21.4, 22.8, 25.04, 27.6, 30.2, 34.1, 40.6, 51.1, 52.4, 68.4, 115.8 ($^{2}J_{C\text{-}F}$ = 22 Hz), 127.7 ($^{3}J_{C\text{-}F}$ = 8.2 Hz), 131.9, 160.1 ($^{2}J_{C\text{-}F}$ = 247 Hz), 173.0, 173.2, 175.8 ppm; IR (KBr): ν 3331, 1745, 1698, 1670, 1511, 1369, 1219, 838 cm^{-1}; HRMS calcd for $C_{19}H_{25}FN_2O_4$ [M + Na]$^+$ 387.1696; Found: 387.1693.

Methyl (1-(3-methoxyphenyl)-2-methyl-5-oxopyrrolidine-2-carbonyl)leucinate (**4l**): Cream solid (yield 218 mg, 58%); mp: 98 – 99 °C; ^{1}H NMR (500 MHz, Chloroform-*d*) δ 0.91 – 0.96 (m, 6H), 1.51 (s, 3H), 1.60 – 1.71 (m, 3H), 2.11 (m, 1H), 2.48 – 2.61 (m, 2H), 2.71 (m, 1H), 3.75 (s, 3H), 3.80 (s, 3H), 4.64 (m, 1H), 6.45 (d, J = 8.3 Hz, 1H), 6.80 – 6.97 (m, 3H), 7.27 (m,

1H) ppm; ^{13}C NMR (126 MHz, Chloroform-*d*) δ 21.4, 21.5, 22.9, 24.9, 30.4, 34.4, 40.6, 51.2, 52.3, 55.4, 68.6, 111.7, 112.4, 117.5, 129.6, 137.1, 160.1, 172.9, 173.5, 175.5 ppm; IR (KBr): ν 3295, 1746, 1673, 1537, 1392, 1213, 1037, 699 cm^{-1}; Anal. calcd for $C_{20}H_{28}N_2O_5$ (376.1998) C, 63.81; H, 7.50; N, 7.44; Found: C, 63.68; H, 7.91; N, 7.10%.

Methyl (1-(4-hydroxyphenyl)-2-methyl-5-oxopyrrolidine-2-carbonyl)leucinate (**4m**): Cream solid (yield, 220 mg, 61%); mp: 165 – 169 °C; ^1H NMR (500 MHz, Chloroform-*d*) δ 0.90 – 0.96 (m, 6H), 1.48 (s, 3H), 1.52 – 1.72 (m, 3H), 2.07 (m, 1H), 2.50 – 2.62 (m, 2H), 2.78 (m, 1H), 3.75 (s, 3H), 4.66 (m, 1H), 6.31 (d, *J* = 8.1 Hz, 1H), 6.63 (d, *J* = 8.8 Hz, 2H), 6.97 (d, *J* = 8.8 Hz, 2H) ppm; ^{13}C NMR (126 MHz, Chloroform-*d*) δ 21.5, 21.7, 22.8, 24.9, 30.3, 33.7, 41.0, 51.2, 51.3, 68.7, 116.3, 127.4, 155.8, 173.0, 173.2, 176.7 ppm; IR (KBr): ν 3600 – 2600 (–OH and –NH), 1746, 1681, 1644, 1517, 1391, 1275, 1228, 833 cm^{-1}; HRMS calcd for $C_{19}H_{26}N_2O_5$ $[M + Na]^+$ 385.1739; Found: 385.1739.

Methyl (2-methyl-5-oxo-1-phenylpyrrolidine-2-carbonyl)phenylalaninate (**4n**): White crystalline solid (yield, 230 mg, 60%); mp: 129 – 130 °C; ^1H NMR (500 MHz, Chloroform-*d*) δ 1.34 (s, 3H), 1.92 (m, 1H), 2.27 (m, 1H), 2.43 (m, 1H), 2.52 (m, 1H), 3.00 (m, 1H), 3.29 (m, 1H), 3.78 (s, 3H), 4.93 (m, 1H), 6.99 (brs, 1H), 7.00 – 7.13 (m, 2H), 7.20 – 7.38 (m, 8H) ppm; ^{13}C NMR (126 MHz, Chloroform-*d*) δ 22.7, 23.0, 30.0, 34.3, 36.7, 53.2, 68.4, 126.0, 126.1, 126.7, 127.1, 128.7, 128.9, 136.0, 136.2, 171.9, 173.8, 175.7 ppm; IR (KBr): ν 3304, 1741, 1692, 1678, 1530, 1381, 1233, 1029, 700 cm^{-1}; Anal. calcd for $C_{22}H_{24}N_2O_4$ (380.1736) C, 69.46; H, 6.36; N, 7.36; Found: C, 69.69; H, 6.50; N, 7.30%.

Methyl (1-(4-chlorophenyl)-2-methyl-5-oxopyrrolidine-2-carbonyl)phenylalaninate (**4o**): Viscous Oil (yield, 224 mg, 54%); ^1H NMR (500 MHz, Chloroform-*d*) δ 1.38 (s, 3H), 2.38 (m, 1H), 2.62 (m, 1H), 2.69 (m, 1H), , 2.75 (m, 1H), 3.02 (m, 1H), 3.30 (m, 1H), 3.82 (s, 3H), 4.93 (m, 1H), 6.61 (d, *J* = 7.9 Hz, 1H, –NH), 6.95 (d, *J* = 7.8 Hz, 1H), 7.02– 7.12 (m, 2H), 7.17 – 7.42 (m, 6H) ppm; ^{13}C NMR (126 MHz, Chloroform-*d*) δ 22.5, 27.6, 29.7, 34.2, 37.7, 53.1, 68.40, 127.18, 127.3, 127.7, 128.8, 129.2, 132.7, 134.4, 135.88, 173.3, 175.7, 176.6 ppm; IR (KBr): ν 3272, 1740, 1652, 1654, 1396, 1223, 704 cm^{-1}; Anal. calcd for $C_{22}H_{23}ClN_2O_4$ (414.1346) C, 63.69; H, 5.59; N, 6.75; Found: C, 63.30; H, 5.72; N, 6.40%.

Methyl (1-(4-hydroxyphenyl)-2-methyl-5-oxopyrrolidine-2-carbonyl)phenylalaninate (**4p**): Cream solid (yield, 218 mg, 55%); mp: 144 – 147°C; ^1H NMR (500 MHz, Chloroform-*d*) δ 1.36 (s, 3H), 1.99 (m, 1H), 2.10 (m, 1H), 2.34 – 2.55 (m, 2H), 3.05 (m, 1H), 3.23 (m, 1H), 3.77 (s, 3H), 4.87 (m, 1H), 6.36 (d, *J* = 7.7 Hz, 1H), 6.77 (m, 2H), 6.82 – 7.20 (m, 5H), 7.23 – 7.32 (m, 3H) ppm; ^{13}C NMR (126 MHz, Chloroform-*d*) δ 22.8, 30.0, 33.6, 37.2, 52.5, 53.2, 68.6, 116.4, 127.4, 128.4, 128.6, 128.8, 128.9, 135.5, 155.7, 171.7, 173.0, 176.6 ppm; IR (KBr): ν 3600 – 2500 (OH and NH), 1742, 1685, 1650, 1515, 1387, 1221, 837, 700cm^{-1}; HRMS calcd for $C_{22}H_{24}N_2O_5$ $[M + Na]^+$ 419.1583; Found: 419.1577.

4a: Methyl (2-methyl-5-oxo-1-phenylpyrrolidine-2-carbonyl)tryptophanate

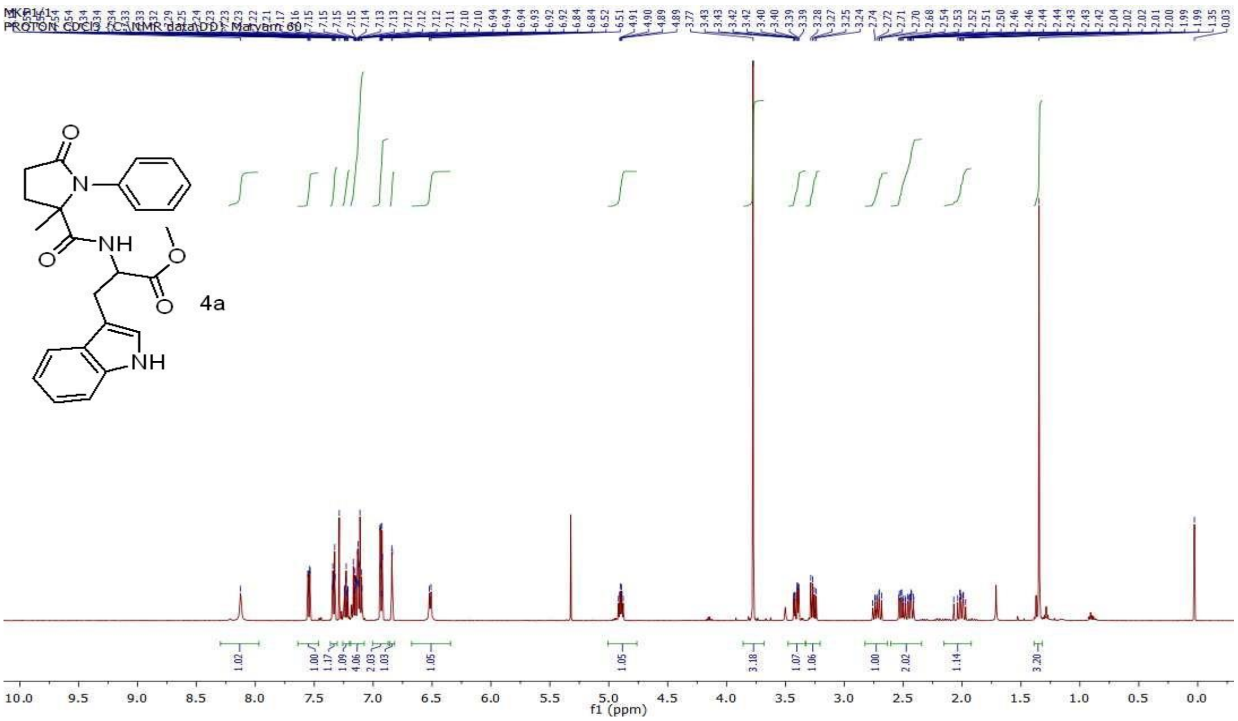

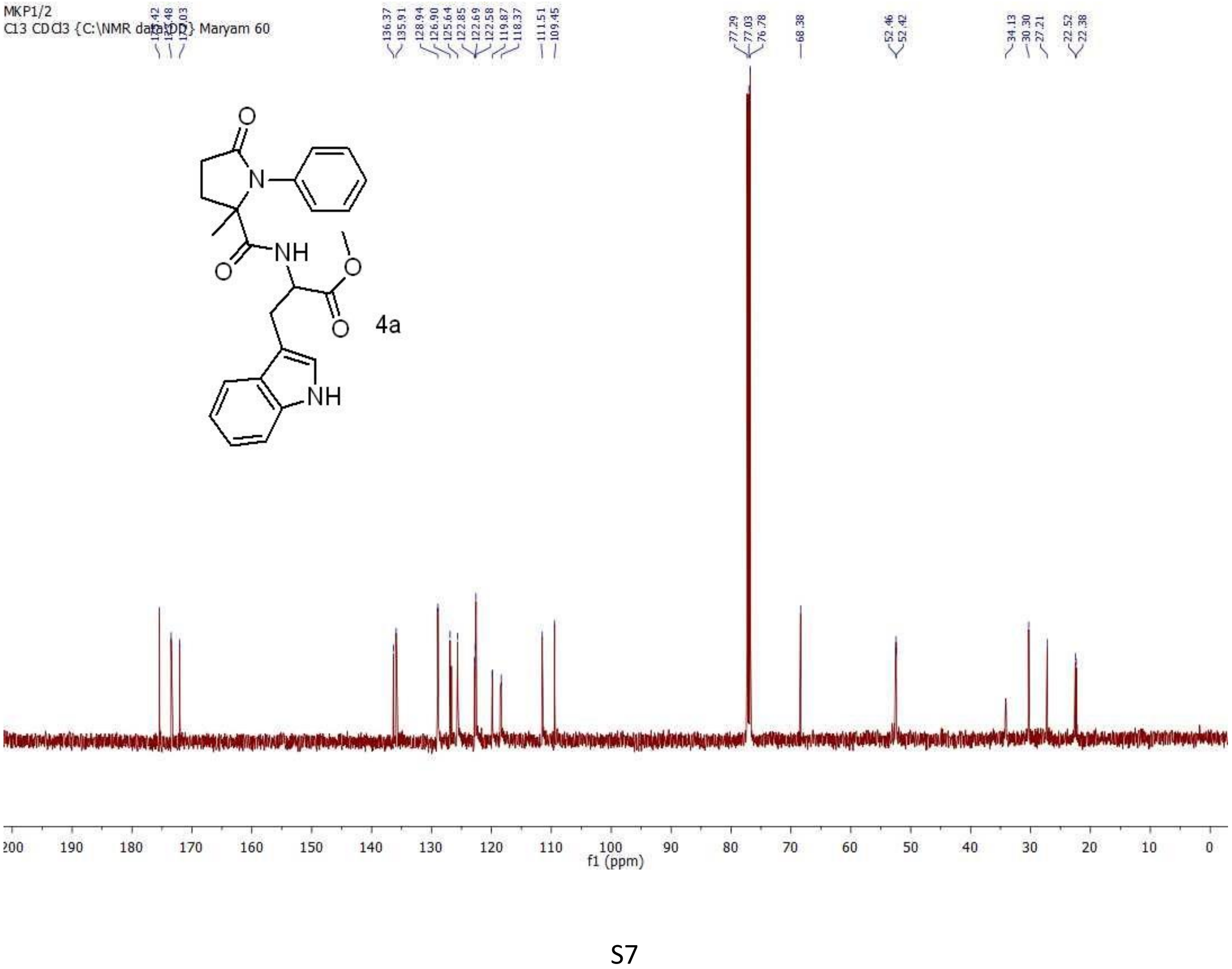

4b: Methyl (1-(4-chlorophenyl)-2-methyl-5-oxopyrrolidine-2-carbonyl)tryptophanate

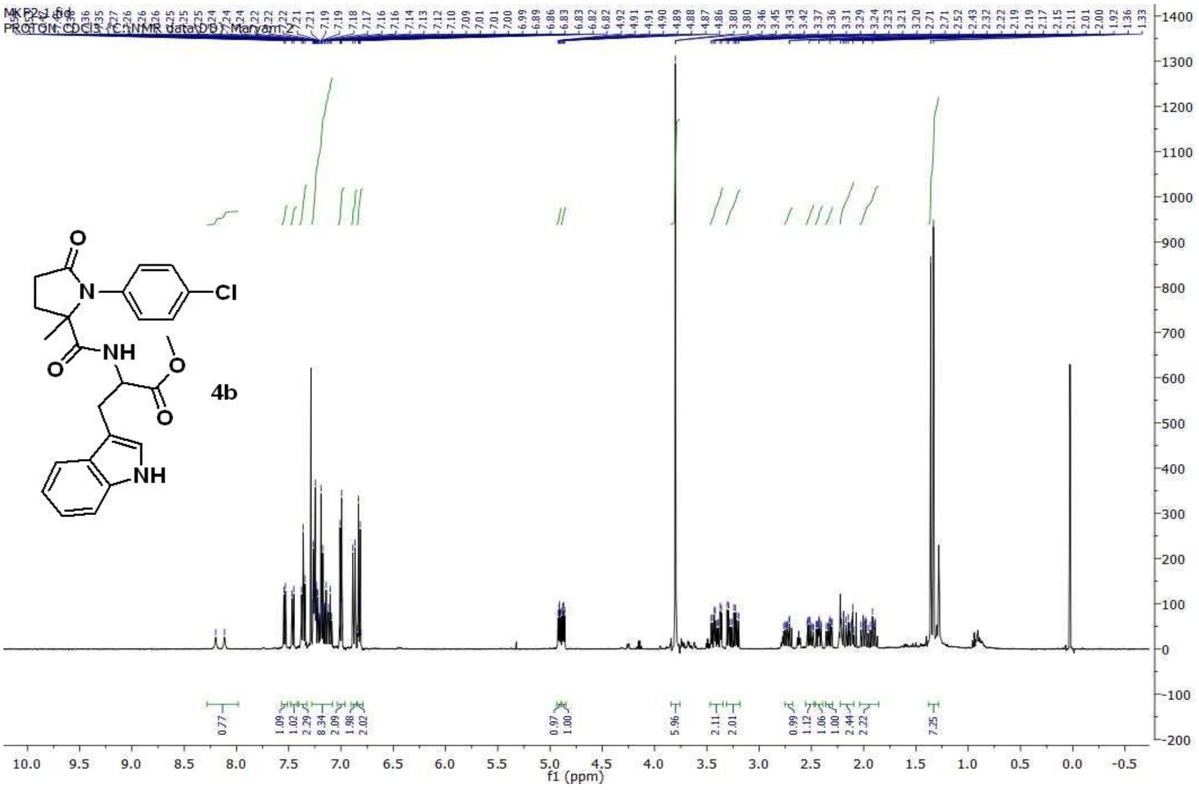

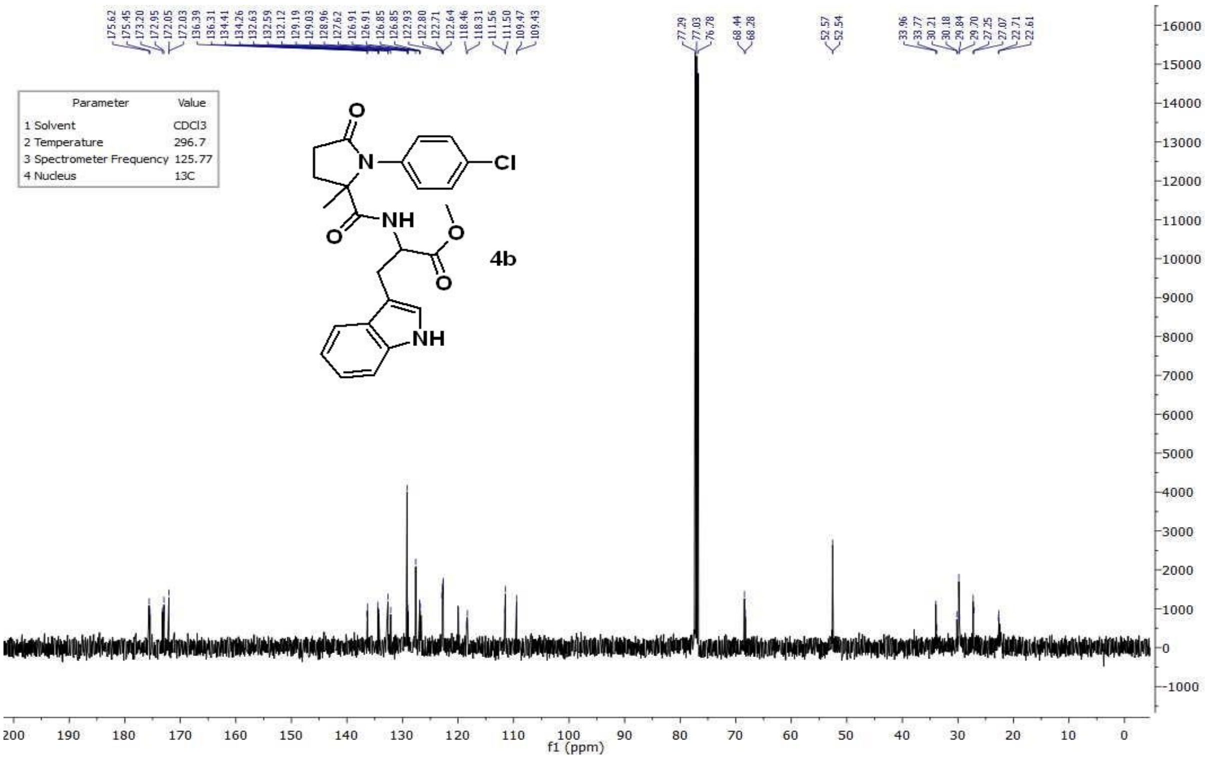

Parameter	Value
1 Solvent	CDCl3
2 Temperature	296.7
3 Spectrometer Frequency	125.77
4 Nucleus	13C

4c: Methyl (1-(2-chlorobenzyl)-2-methyl-5-oxopyrrolidine-2-carbonyl)tryptophanate

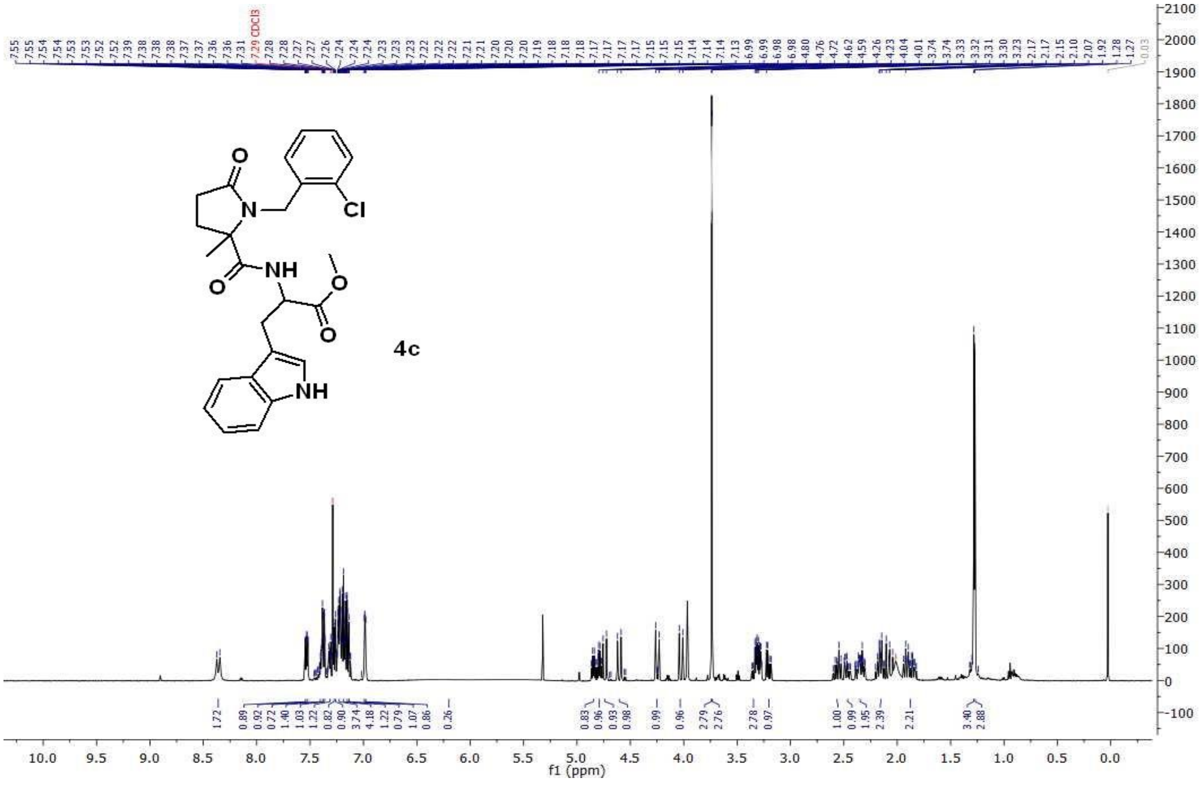

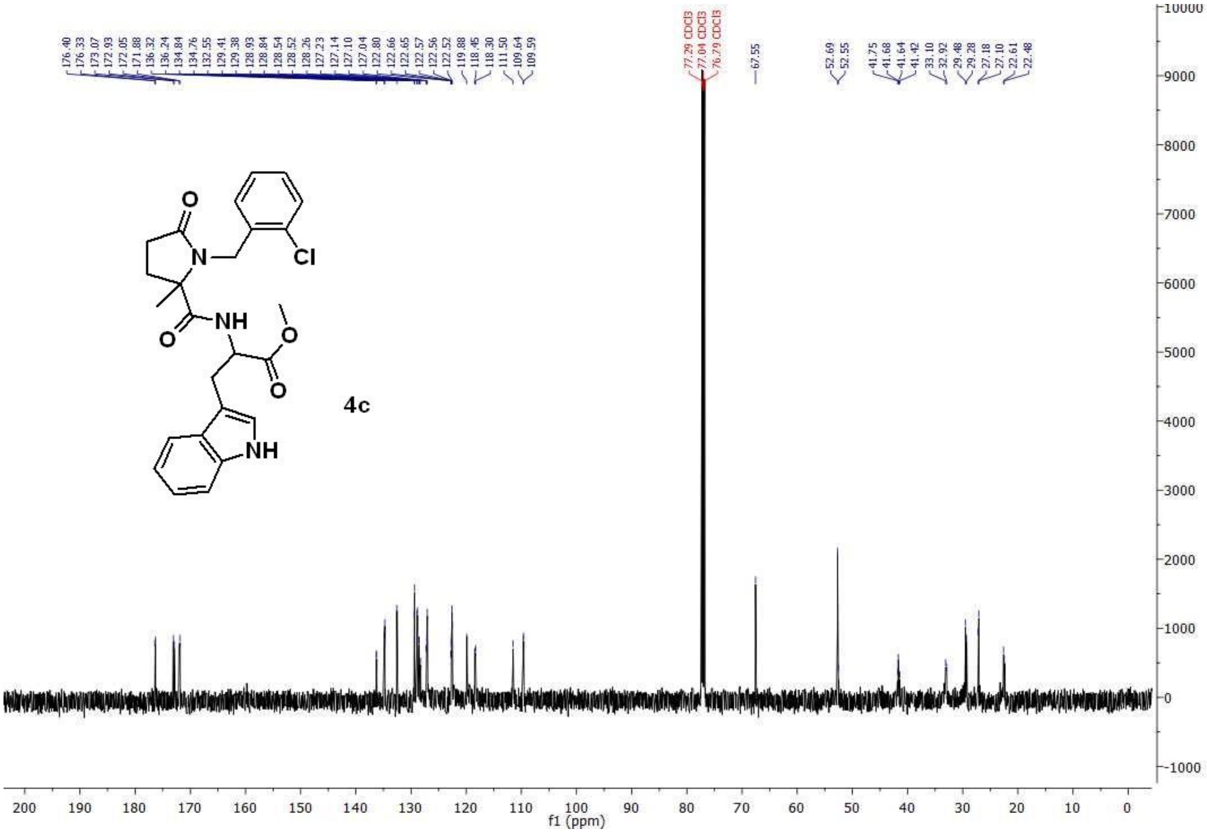

4d: Methyl (1-(4-fluorophenyl)-2-methyl-5-oxopyrrolidine-2-carbonyl)tryptophanate

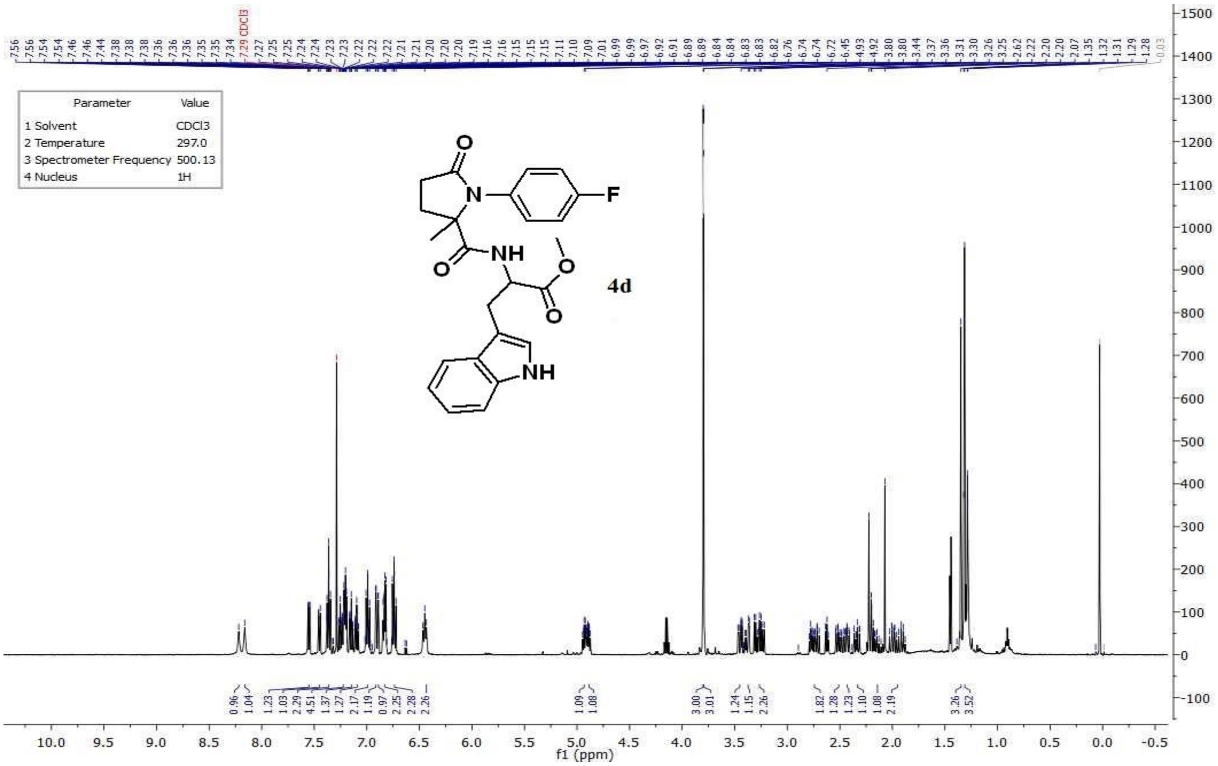

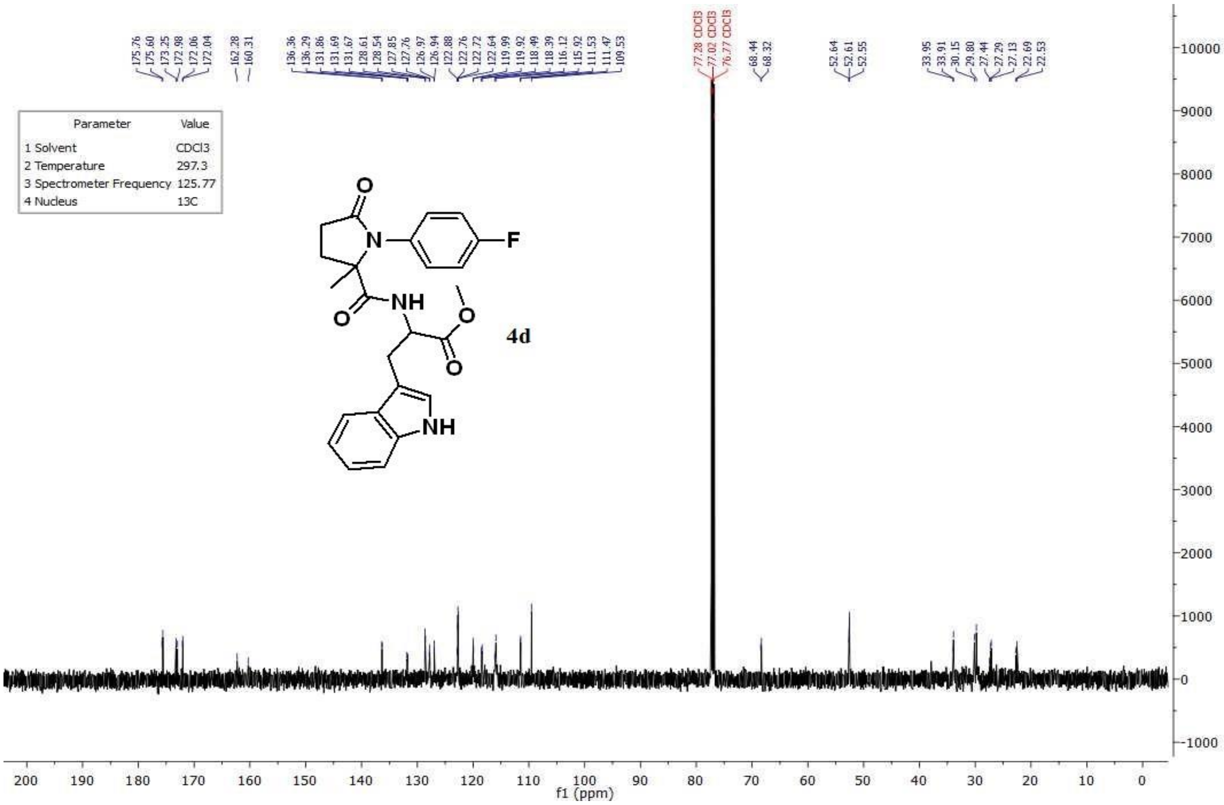

4e: Methyl (1-(3-methoxyphenyl)-2-methyl-5-oxopyrrolidine-2-carbonyl)tryptophanate

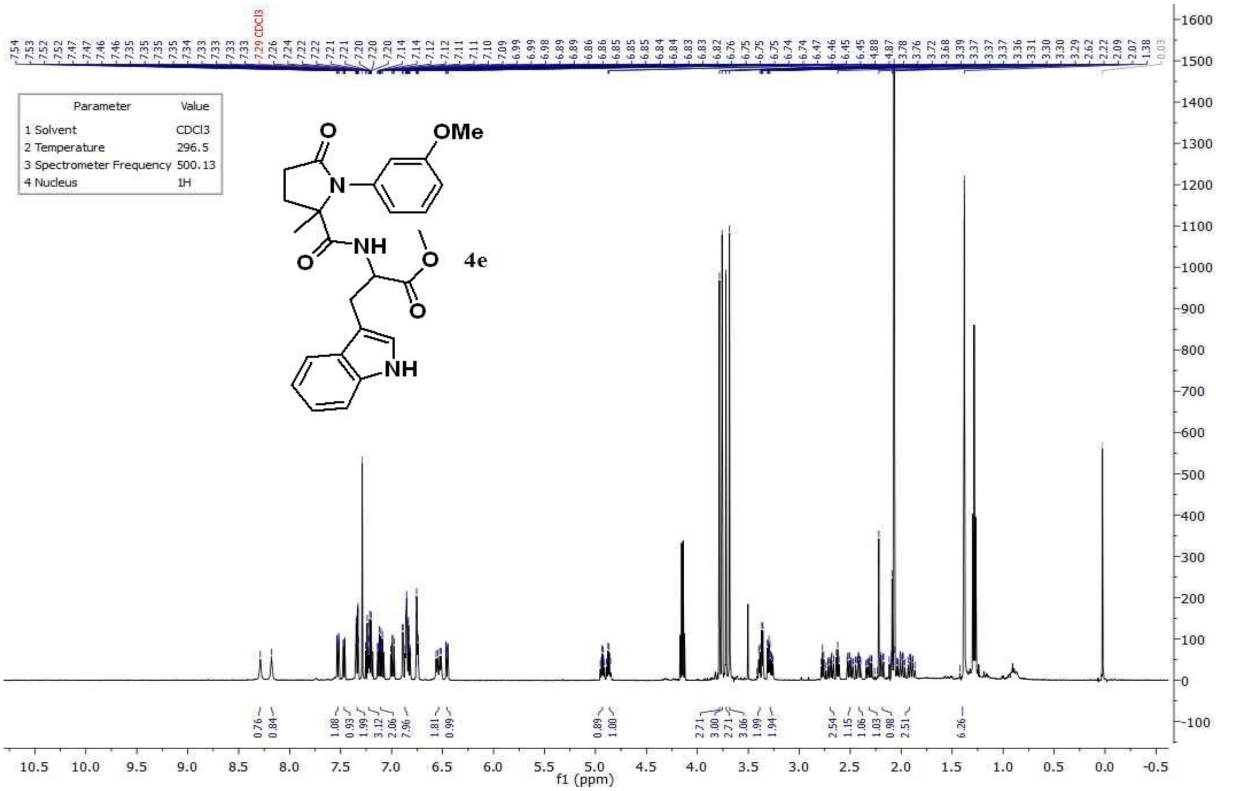

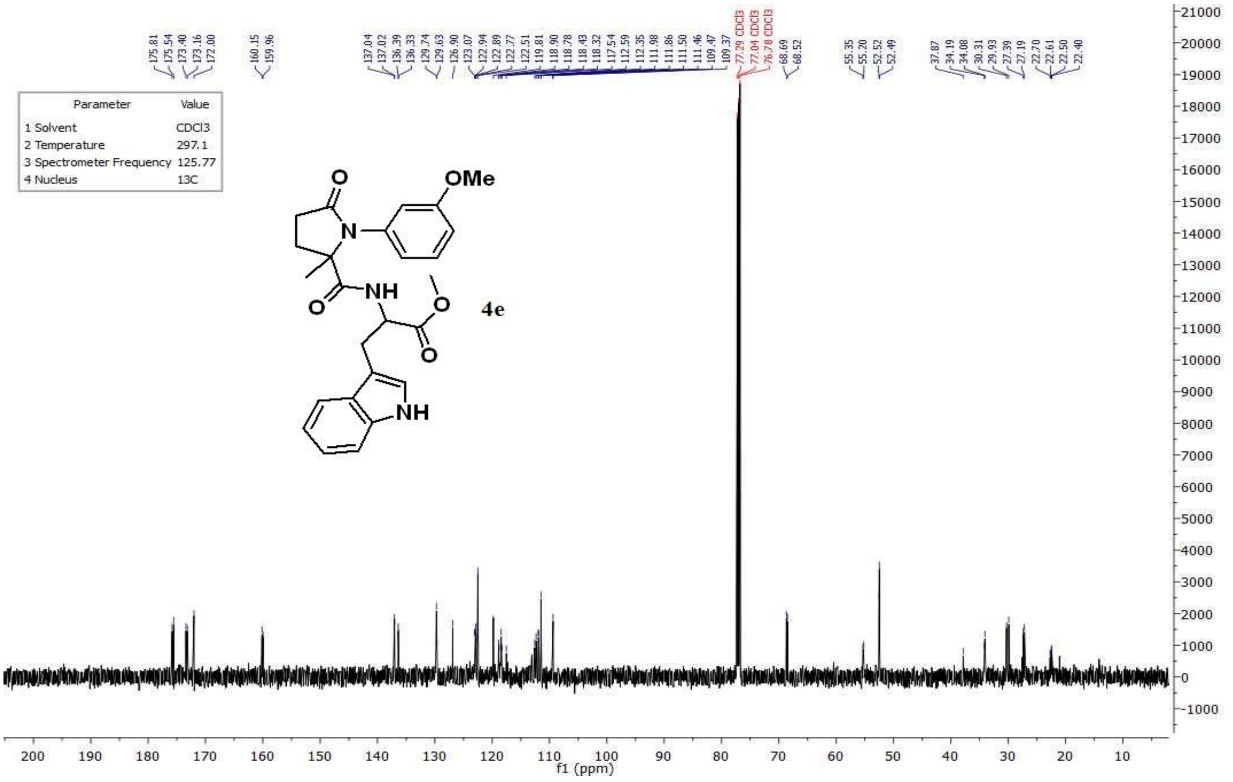

4f: Methyl (2-methyl-5-oxo-1-(*p*-tolyl)pyrrolidine-2-carbonyl)tryptophanate

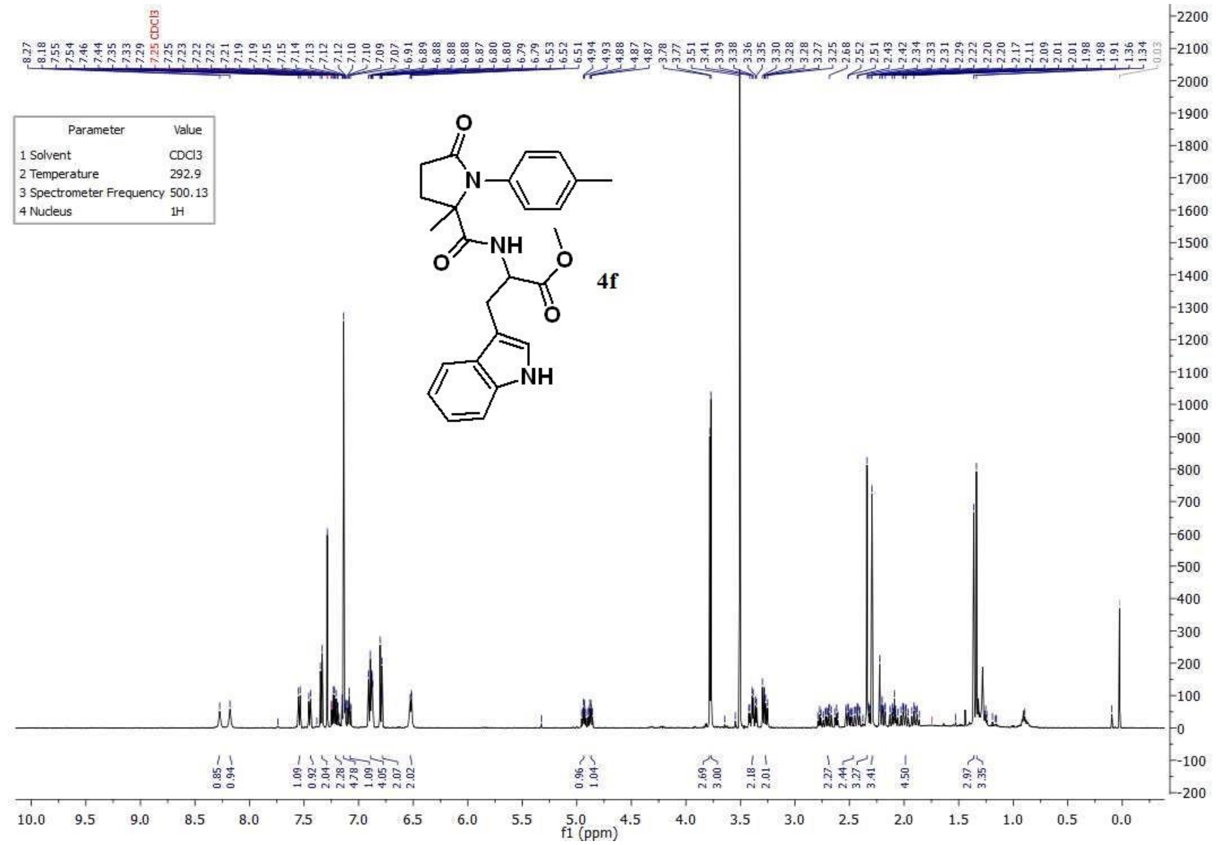

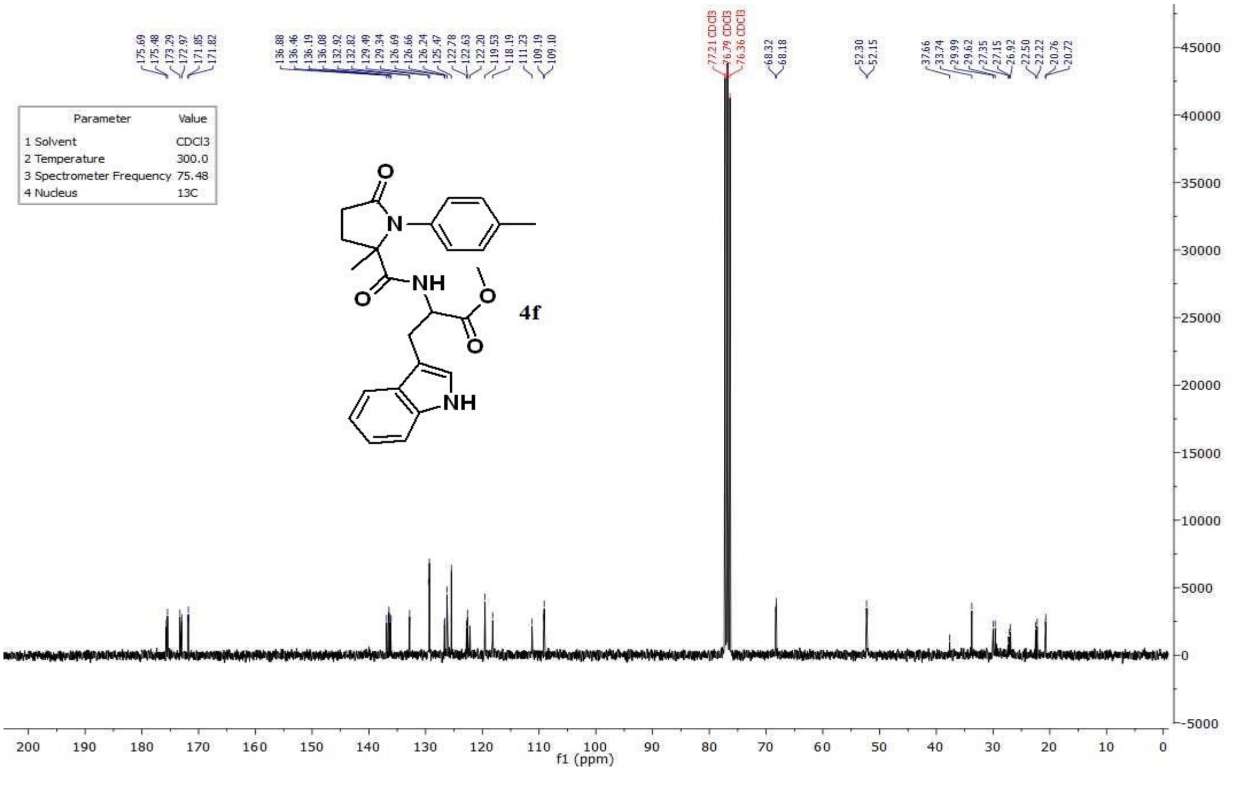

4g: Methyl (2-methyl-5-oxo-1-phenylpyrrolidine-2-carbonyl)leucinate

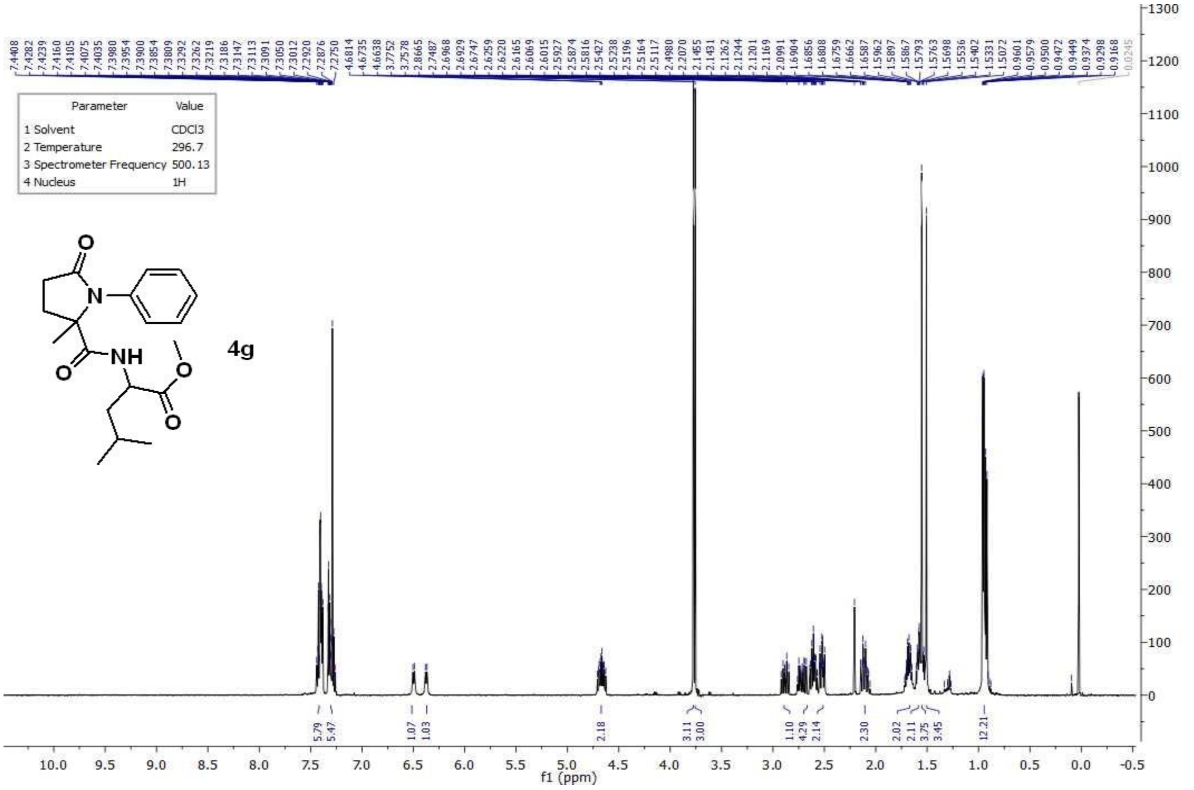

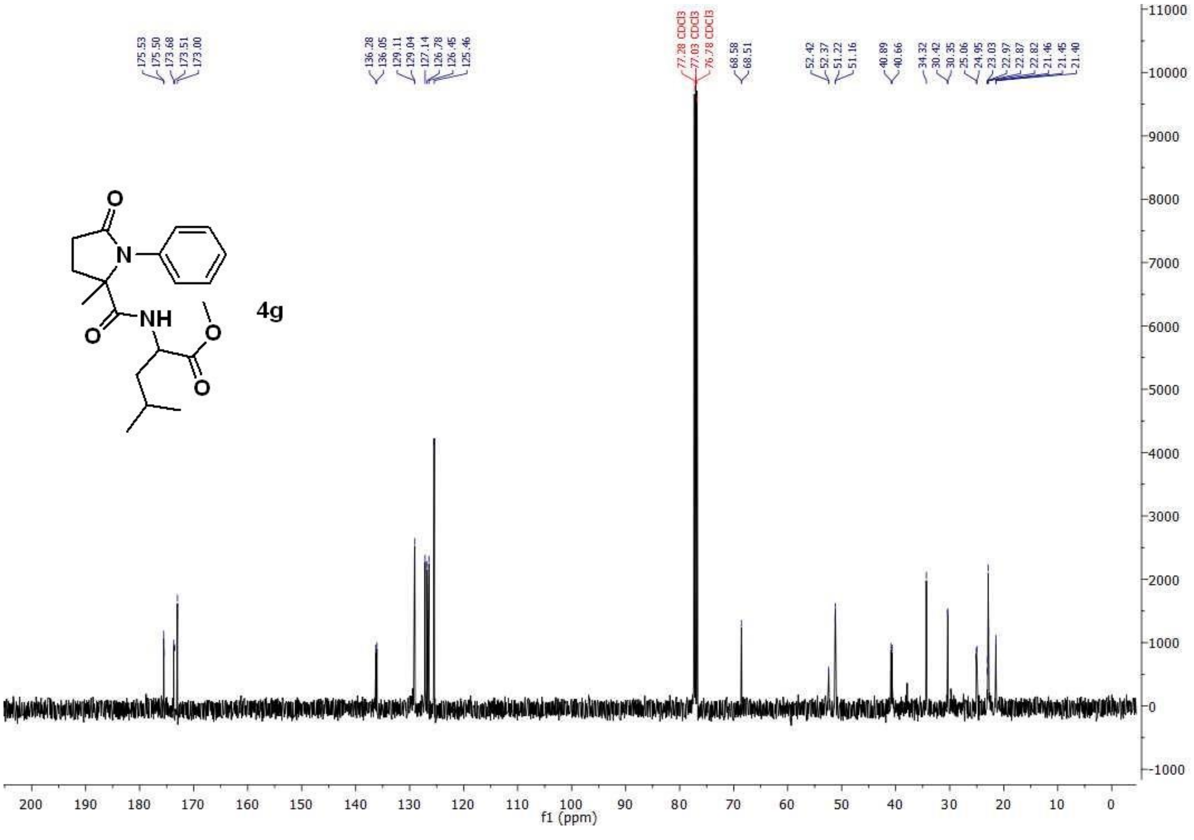

4h: Methyl (1-(4-chlorophenyl)-2-methyl-5-oxopyrrolidine-2-carbonyl)leucinate

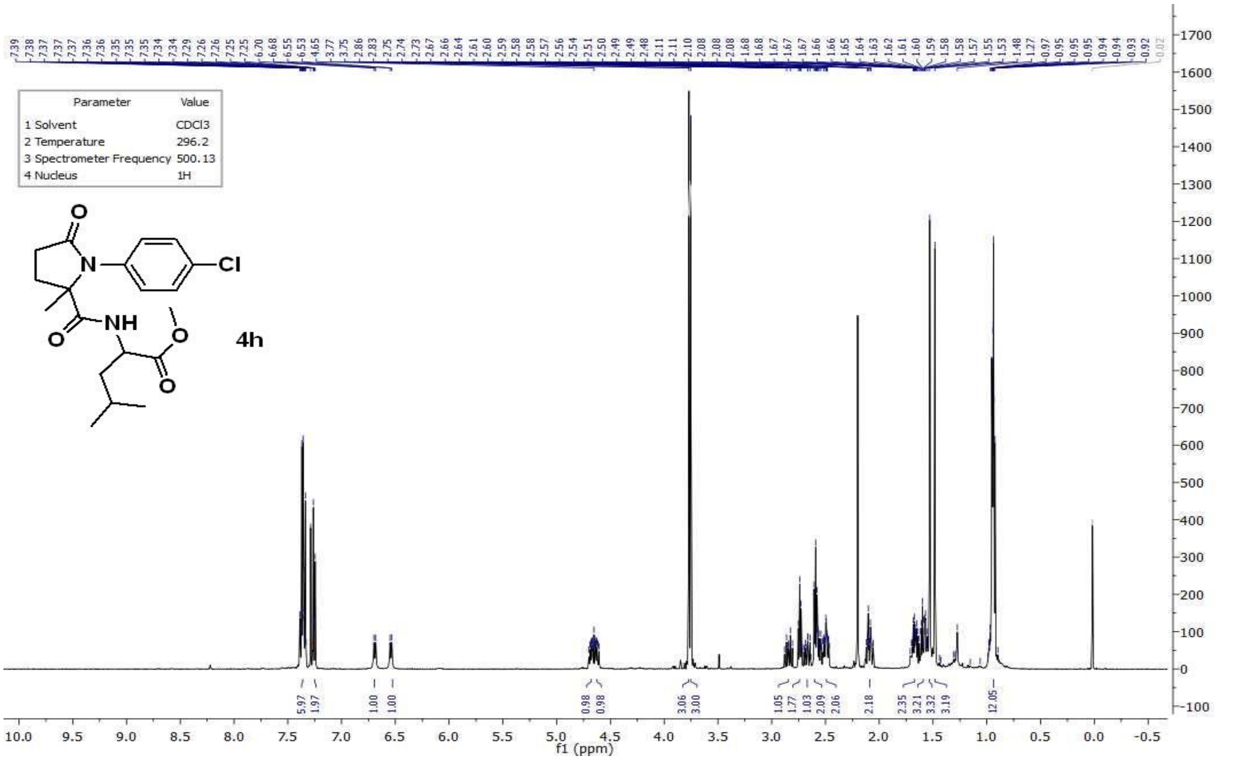

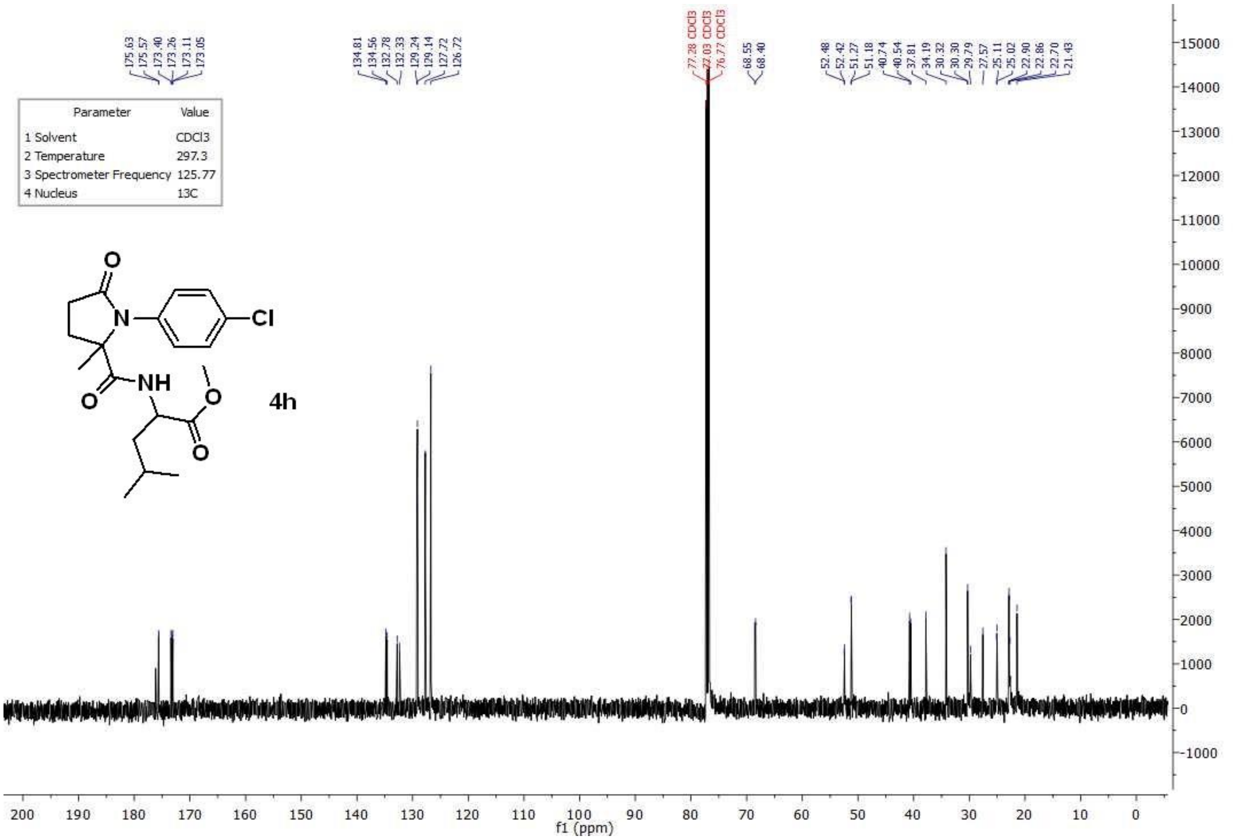

4i: Methyl (2-methyl-5-oxo-1-(p-tolyl)pyrrolidine-2-carbonyl)leucinate

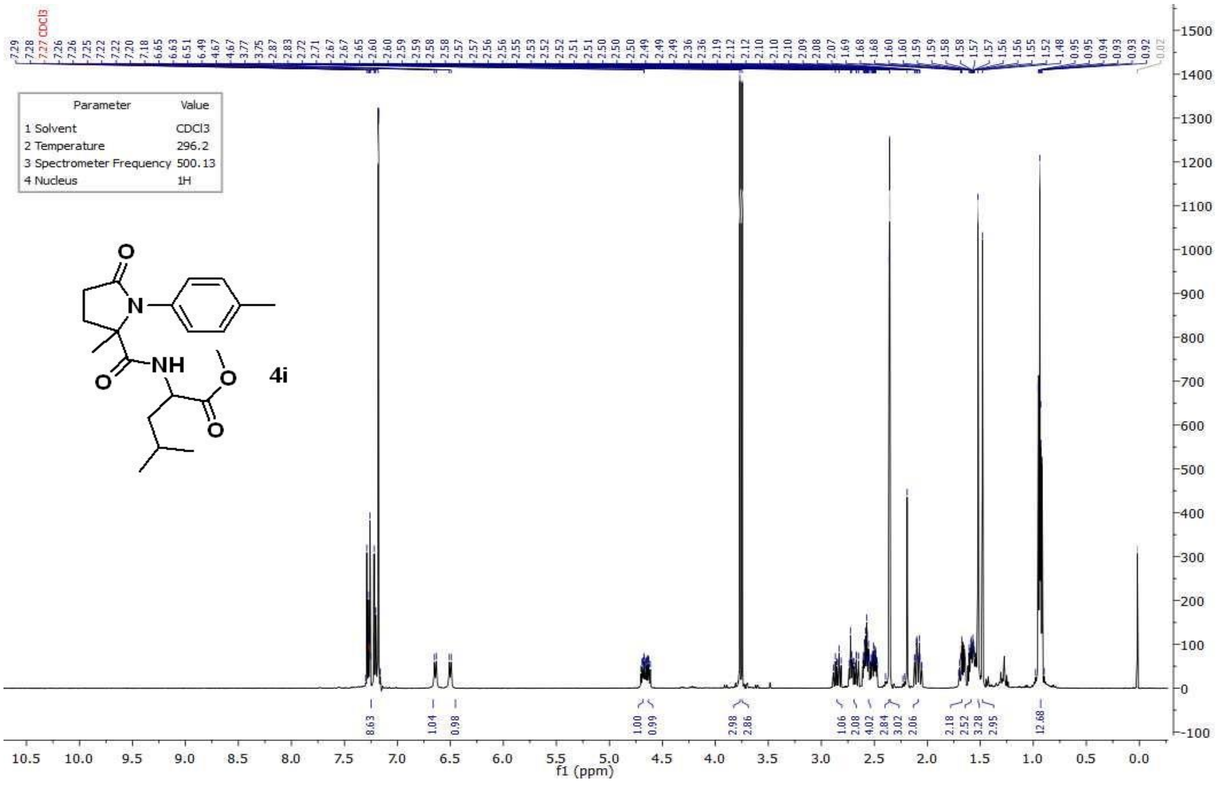

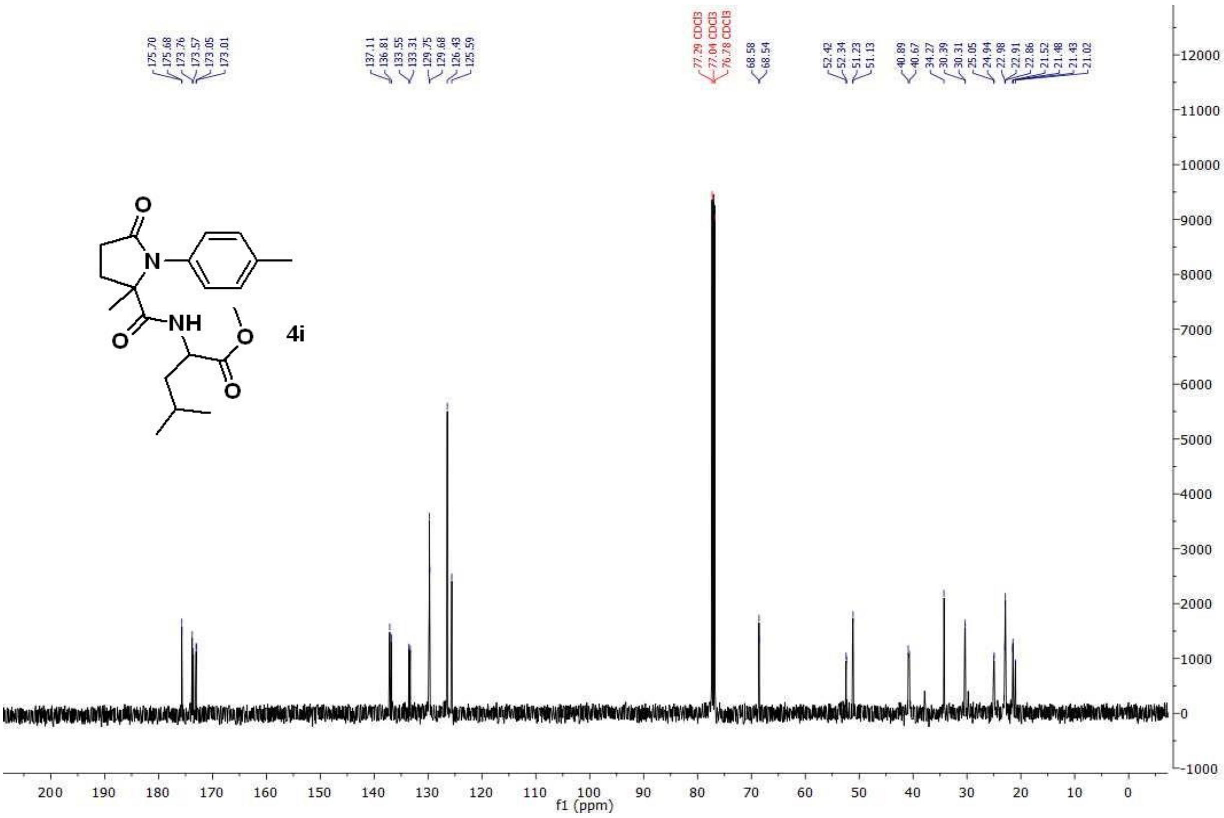

4j: Methyl (1-(2-(1H-indol-3-yl)ethyl)-2-methyl-5-oxopyrrolidine-2-carbonyl)leucinate

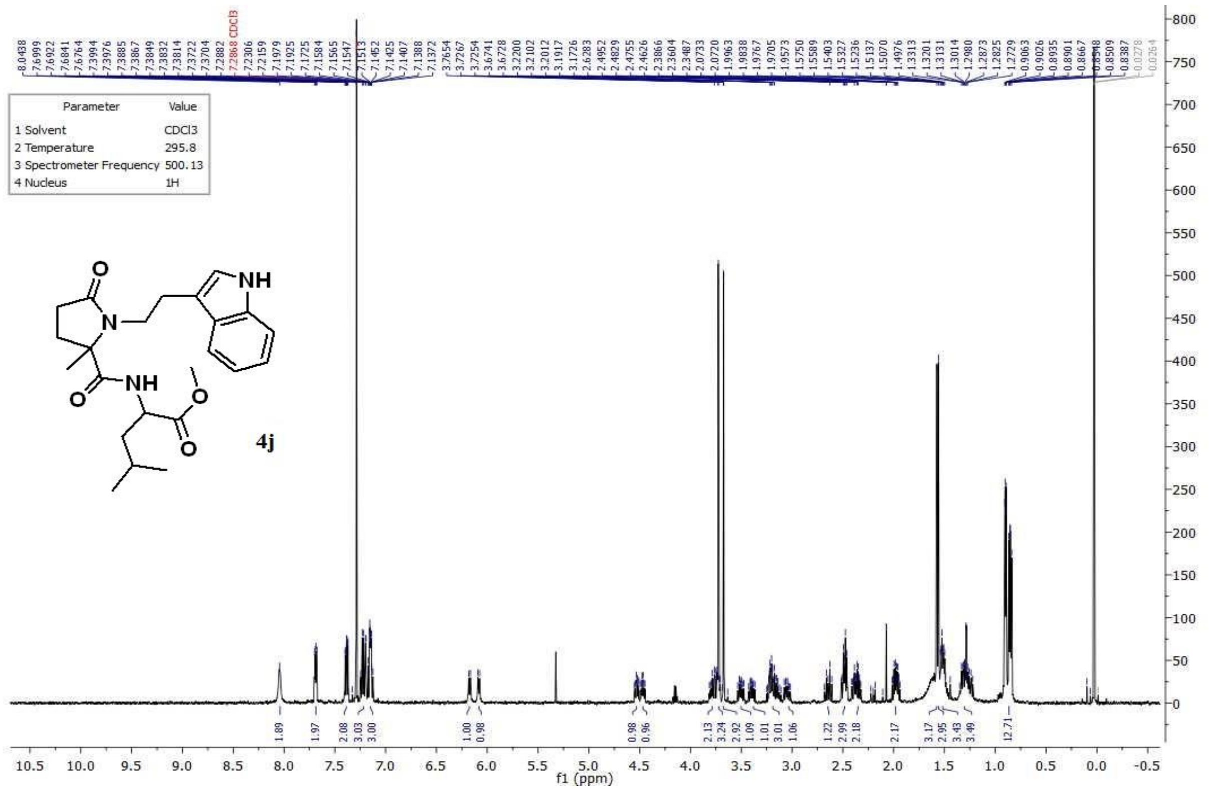

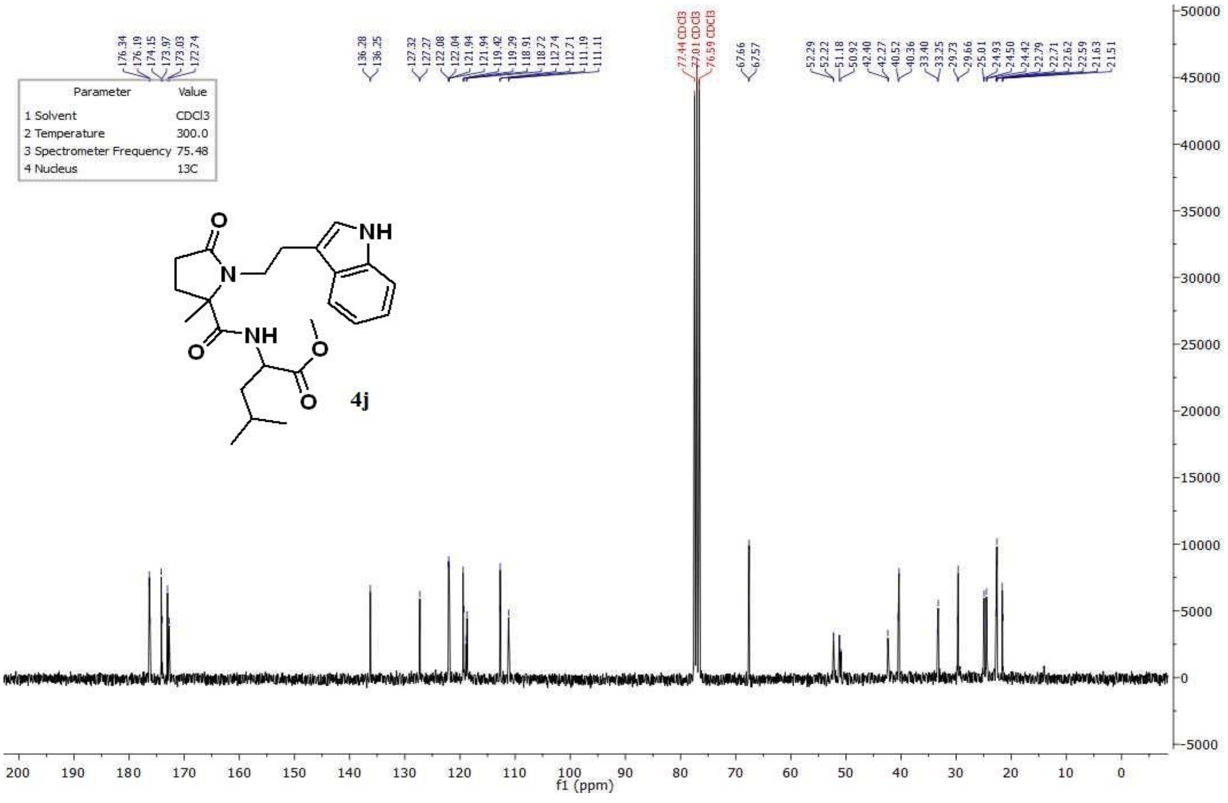

4k: Methyl (1-(4-fluorophenyl)-2-methyl-5-oxopyrrolidine-2-carbonyl)leucinate

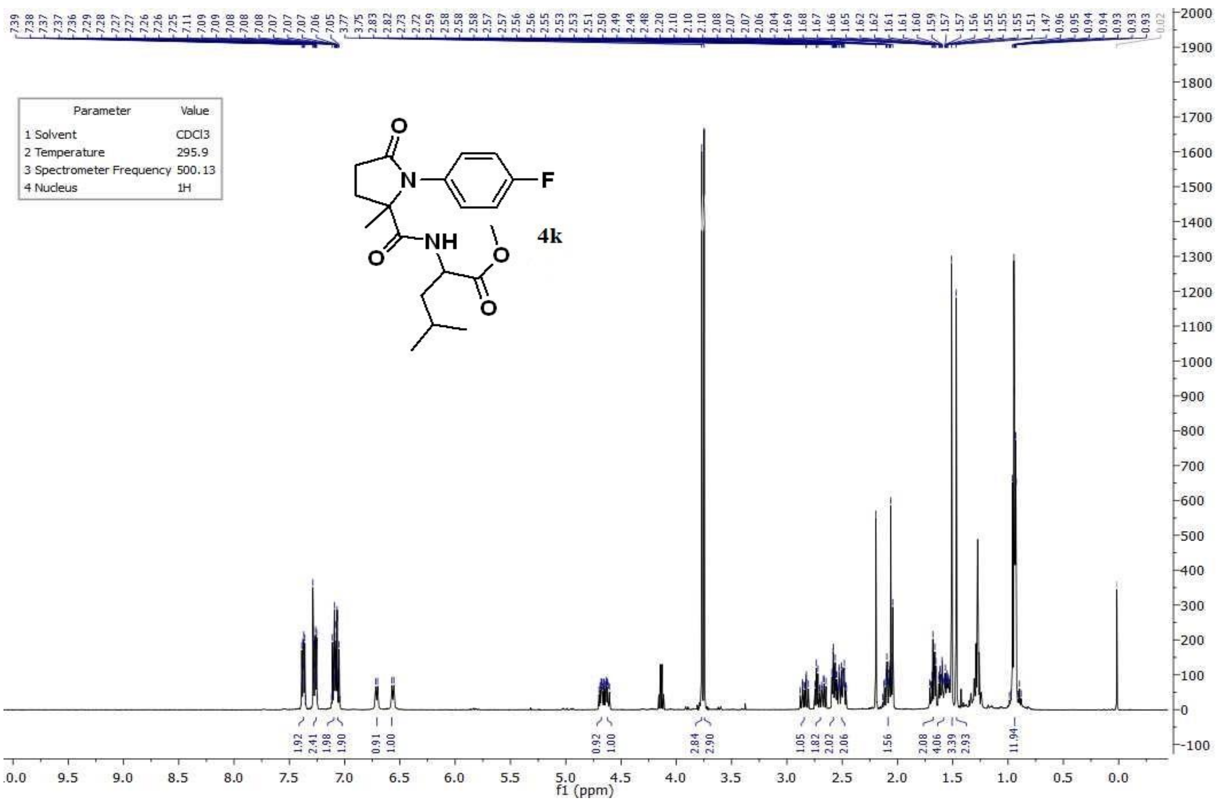

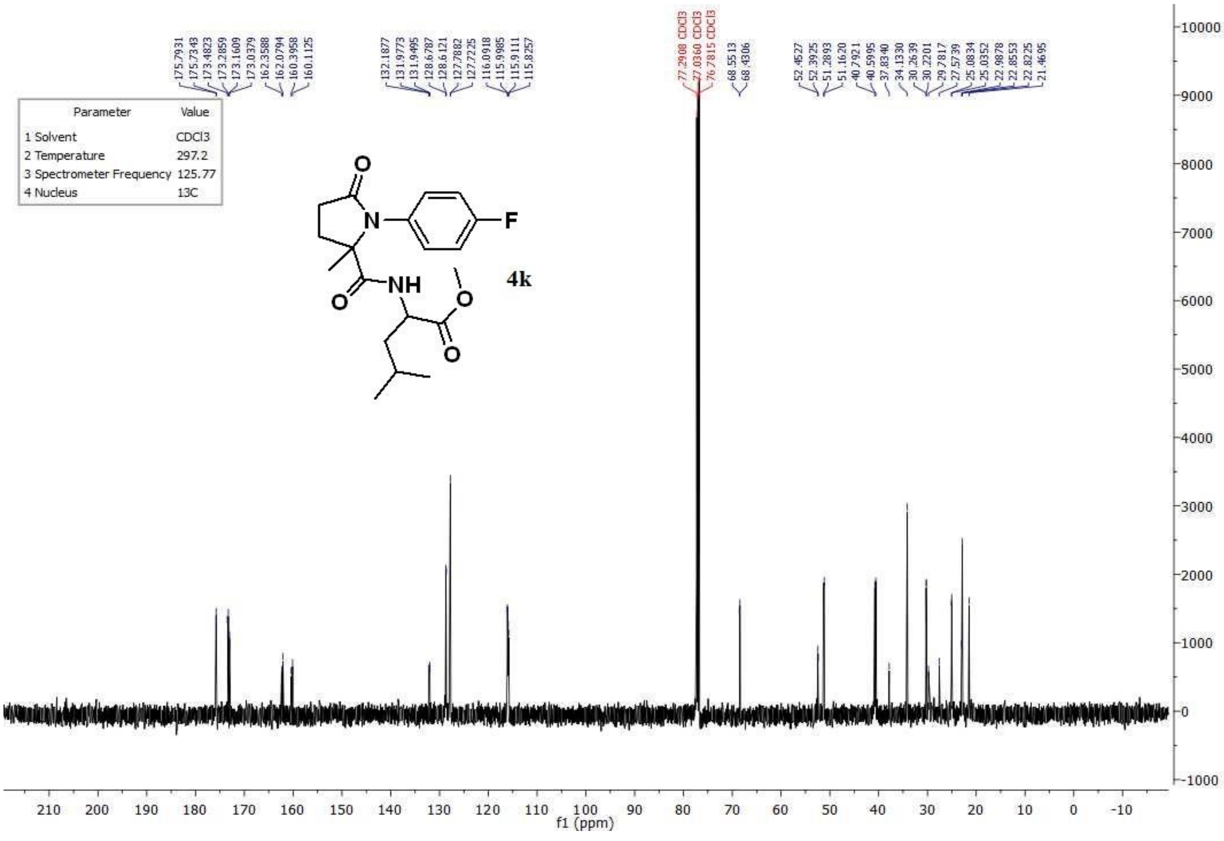

4l: Methyl (1-(3-methoxyphenyl)-2-methyl-5-oxopyrrolidine-2-carbonyl)leucinate

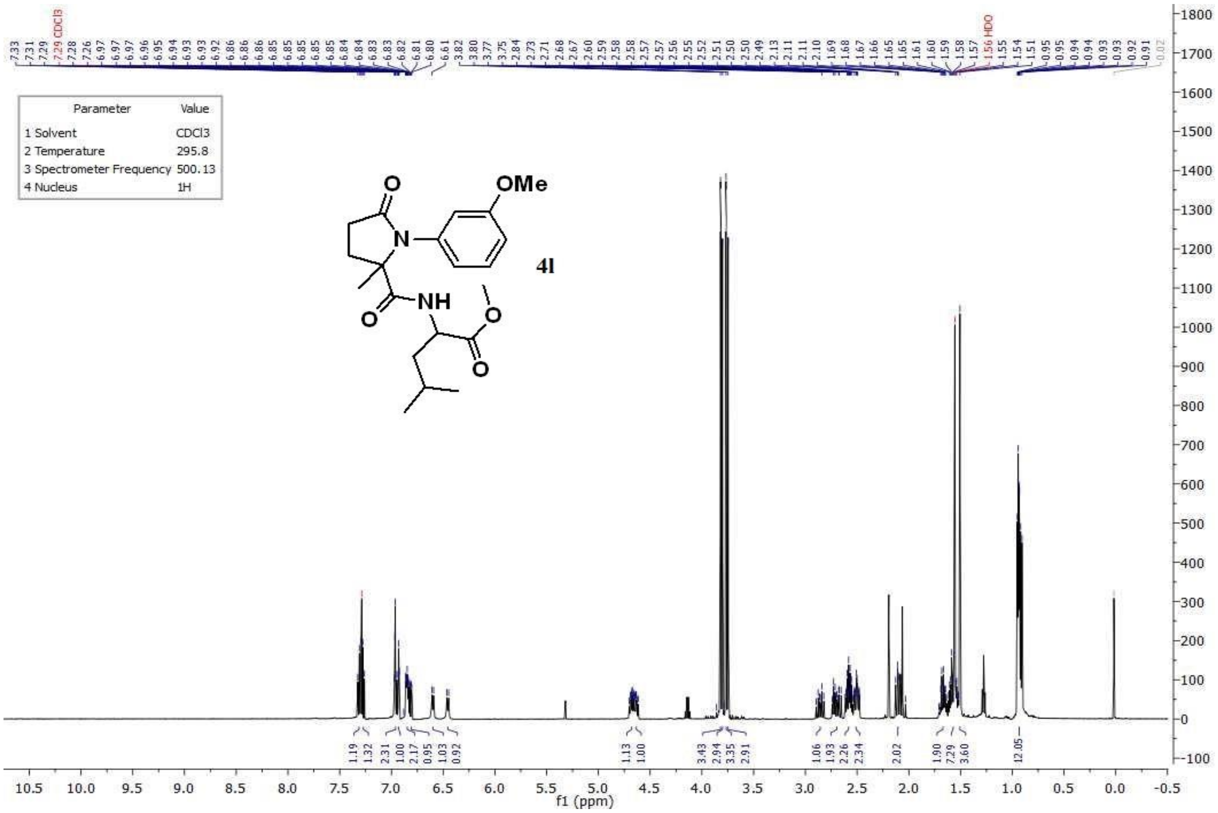

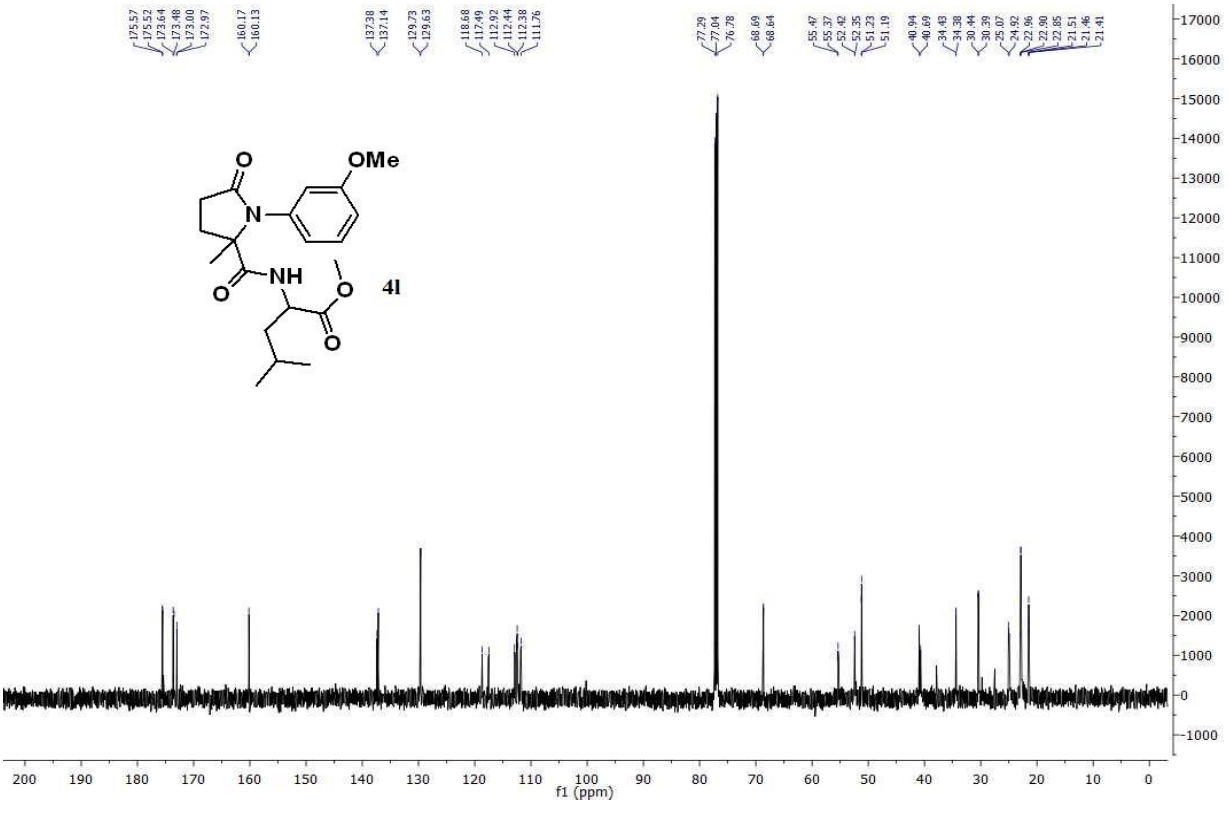

4m: Methyl (1-(4-hydroxyphenyl)-2-methyl-5-oxopyrrolidine-2-carbonyl)leucinate

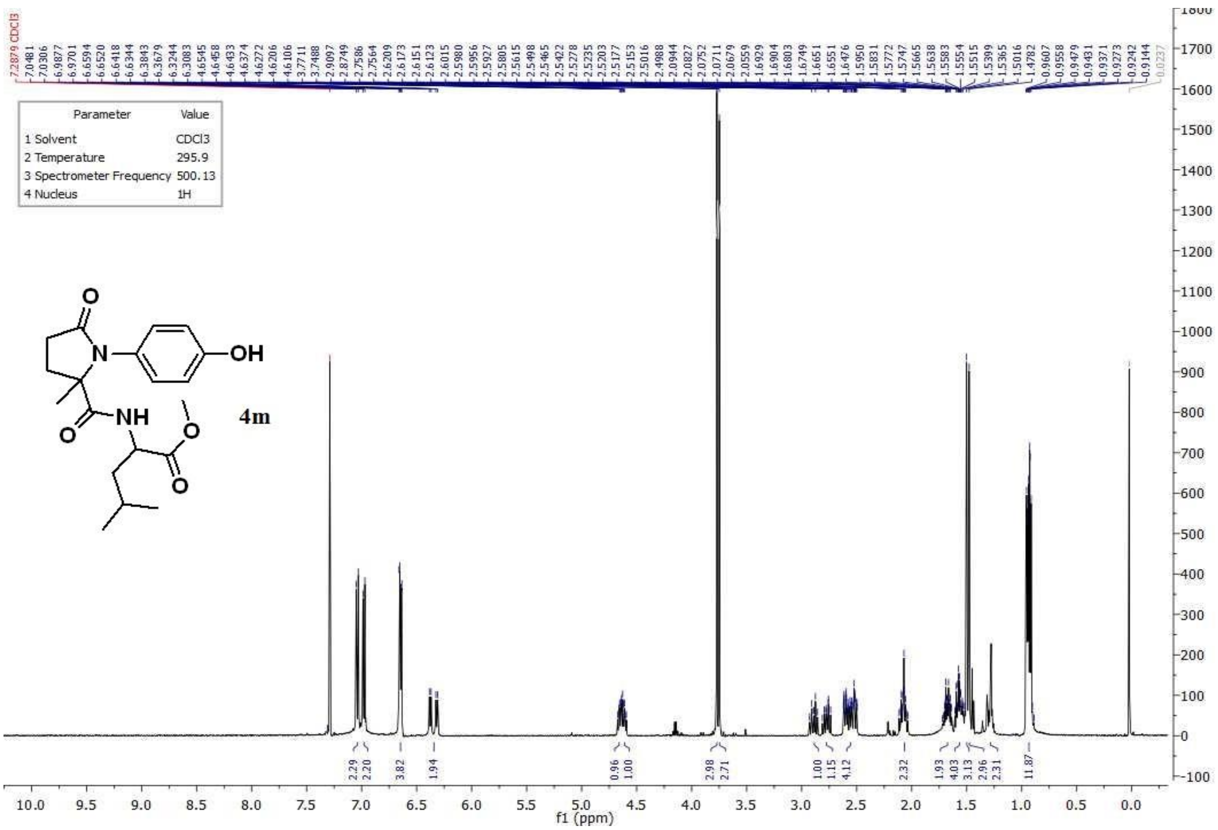

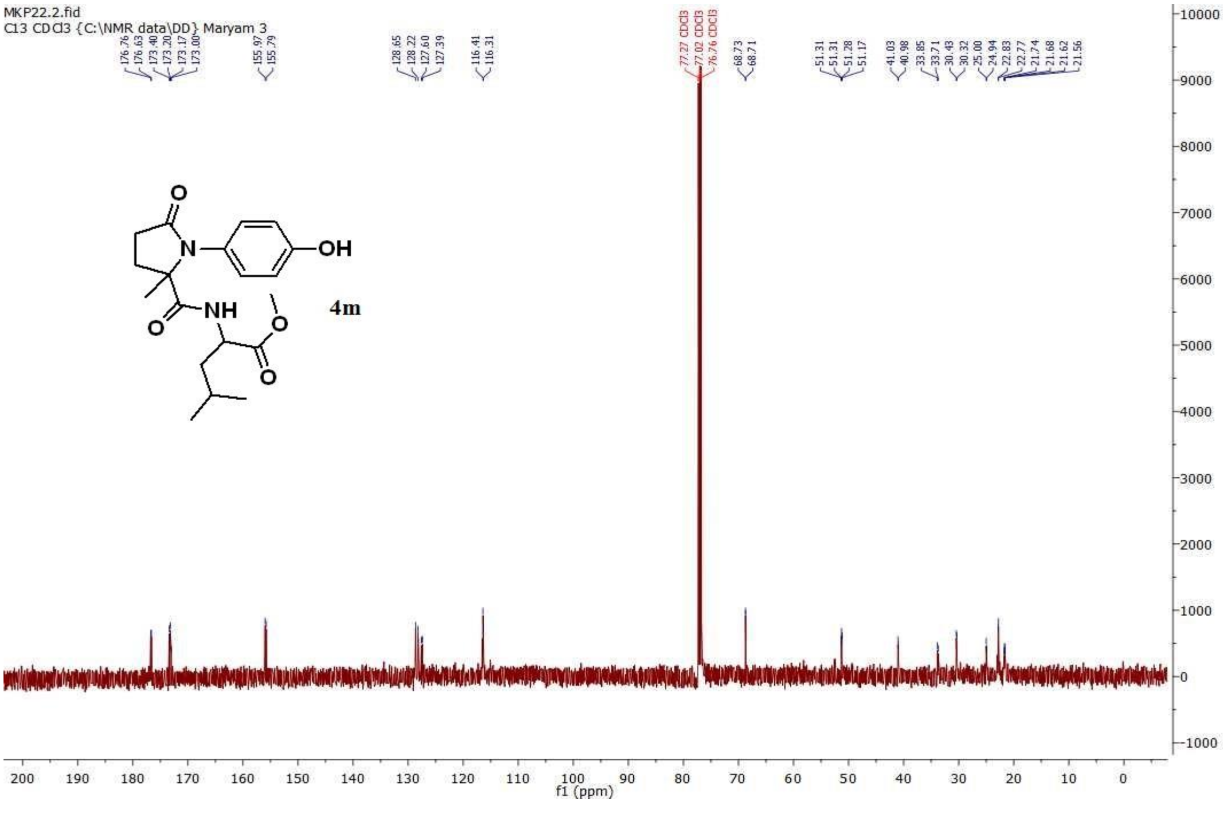

4n: Methyl (2-methyl-5-oxo-1-phenylpyrrolidine-2-carbonyl)phenylalaninate

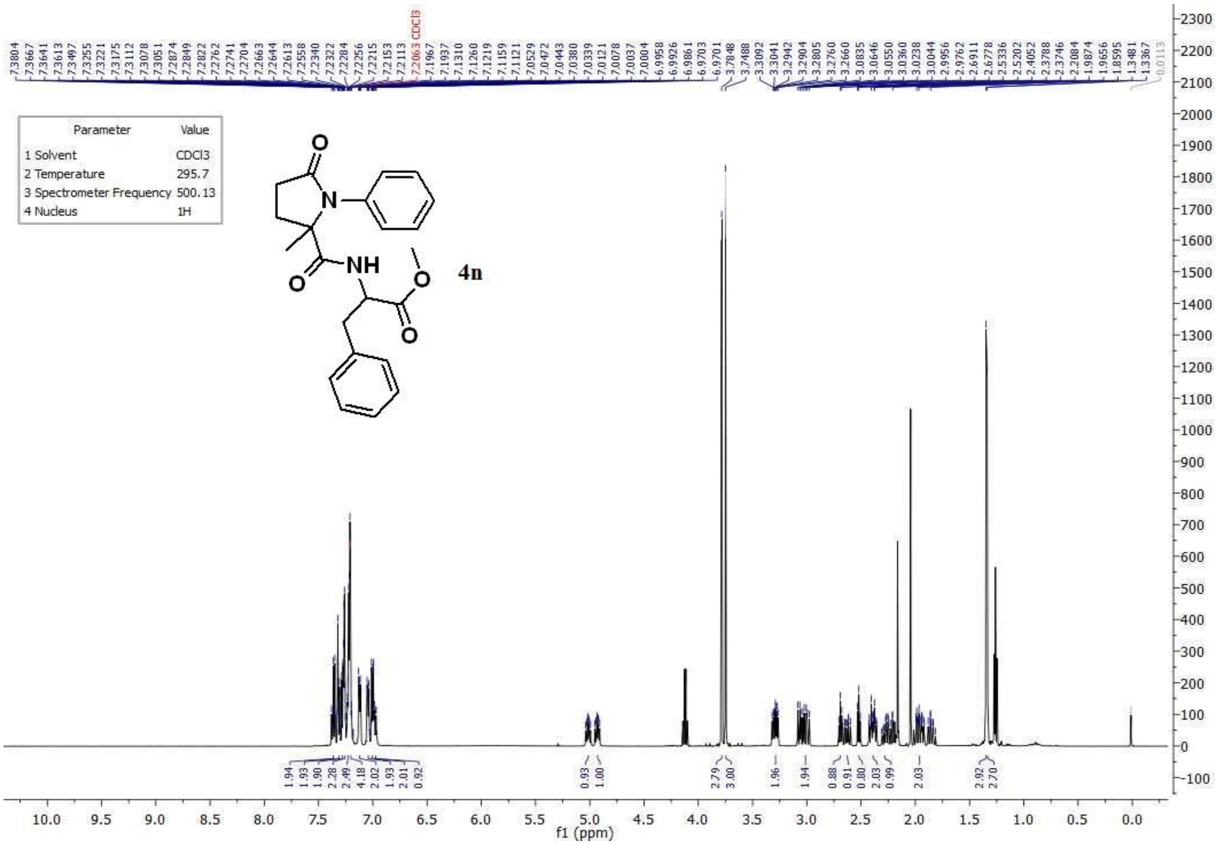

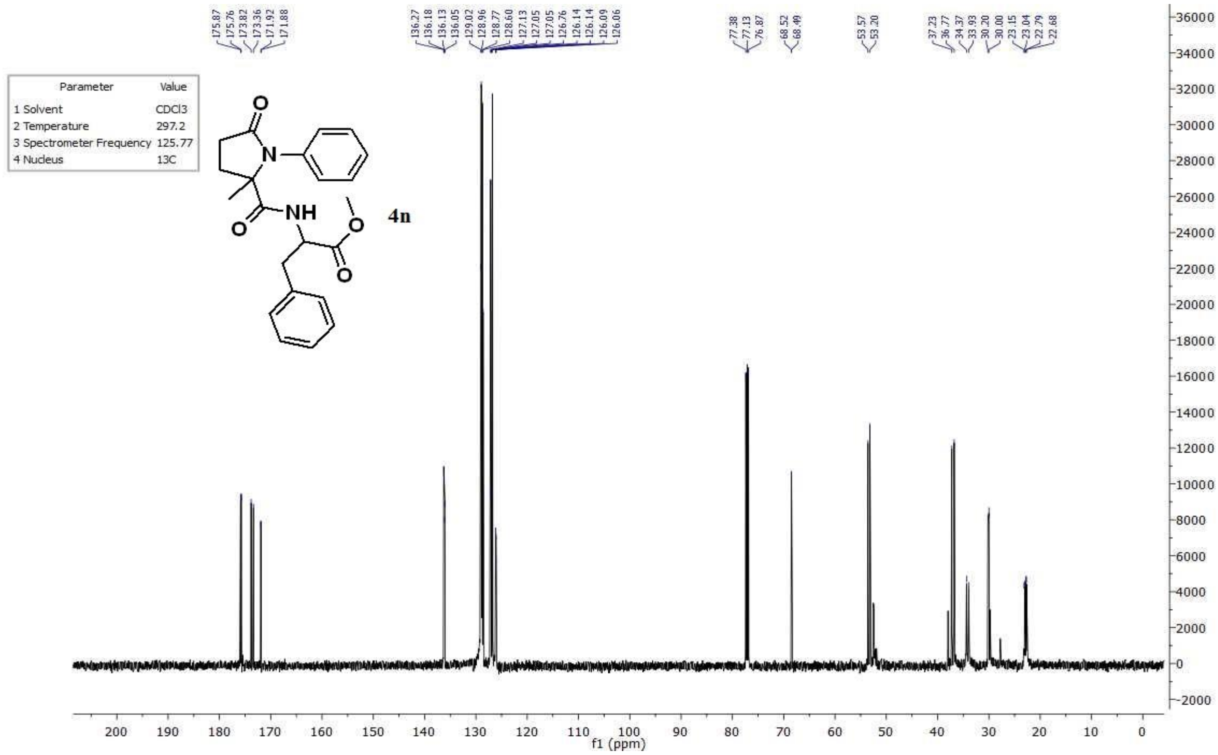

4o: Methyl (1-(4-chlorophenyl)-2-methyl-5-oxopyrrolidine-2-carbonyl)phenylalaninate

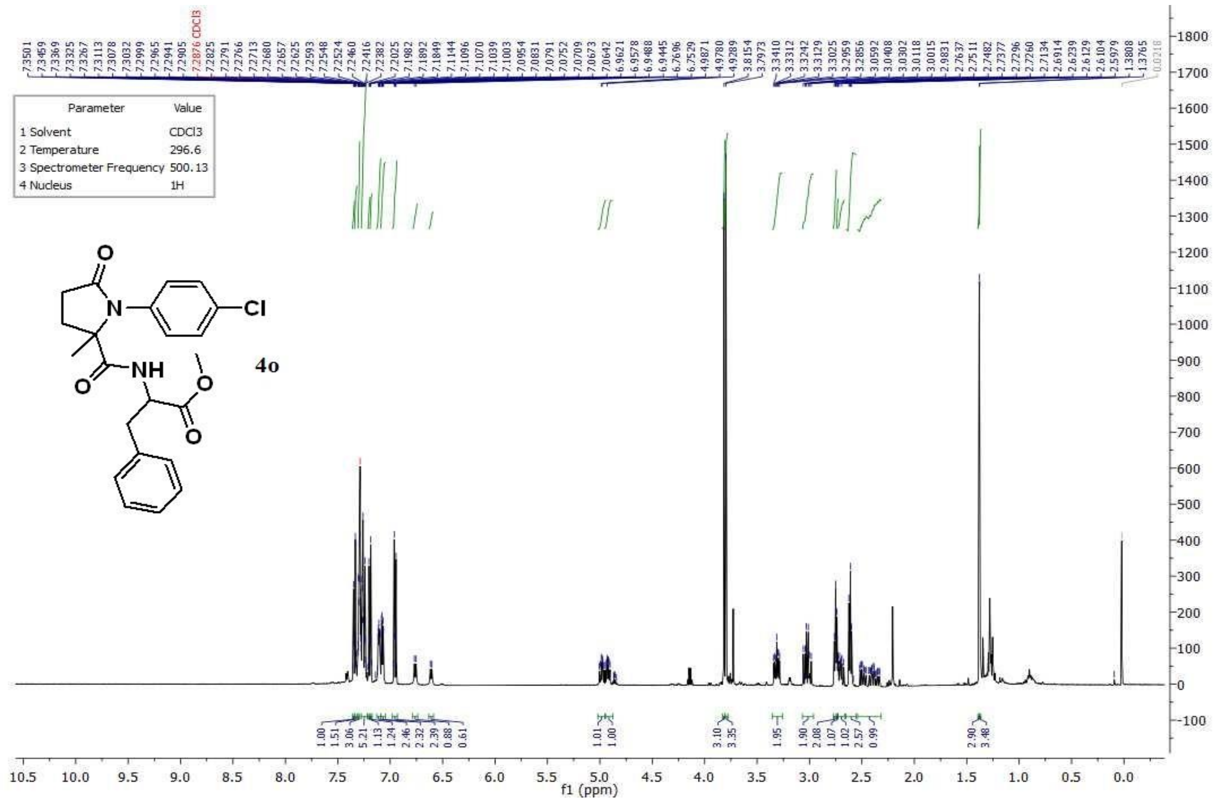

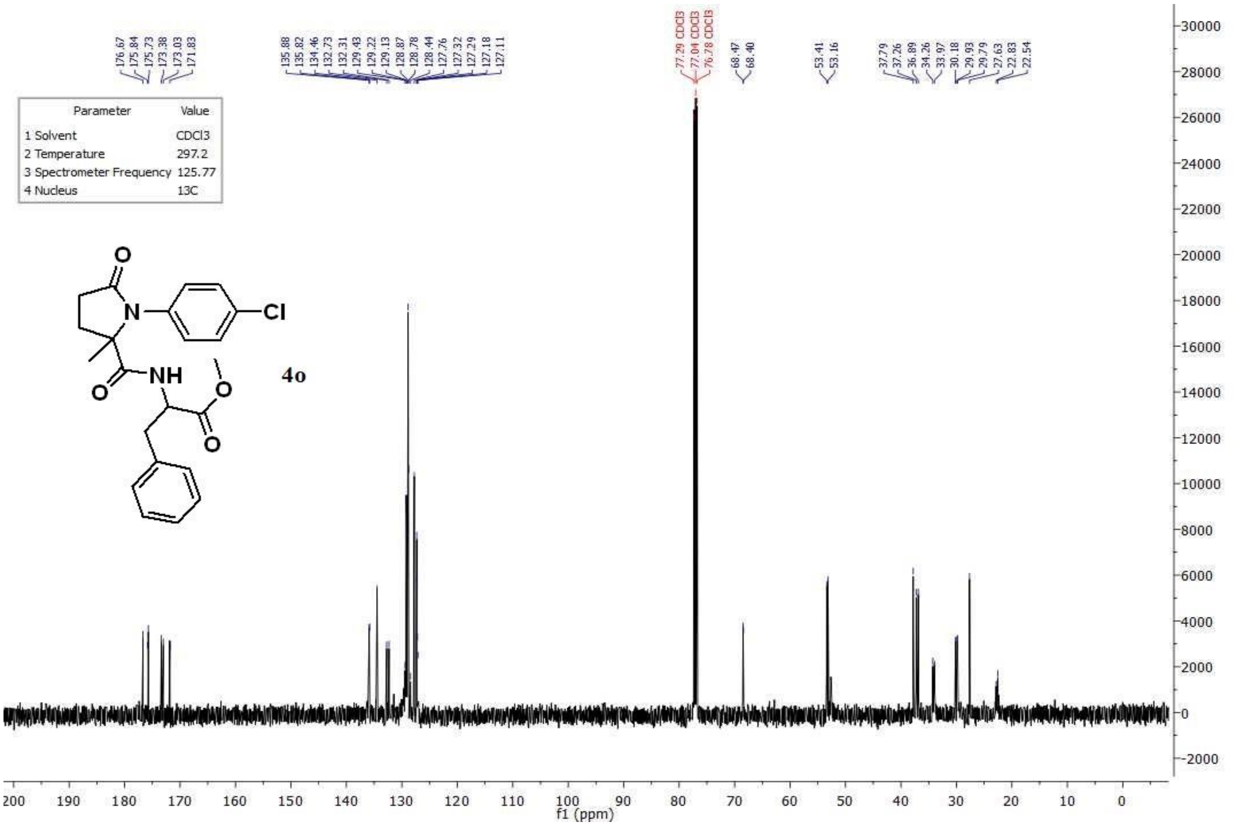

4p: Methyl (1-(4-hydroxyphenyl)-2-methyl-5-oxopyrrolidine-2-carbonyl)phenylalaninate

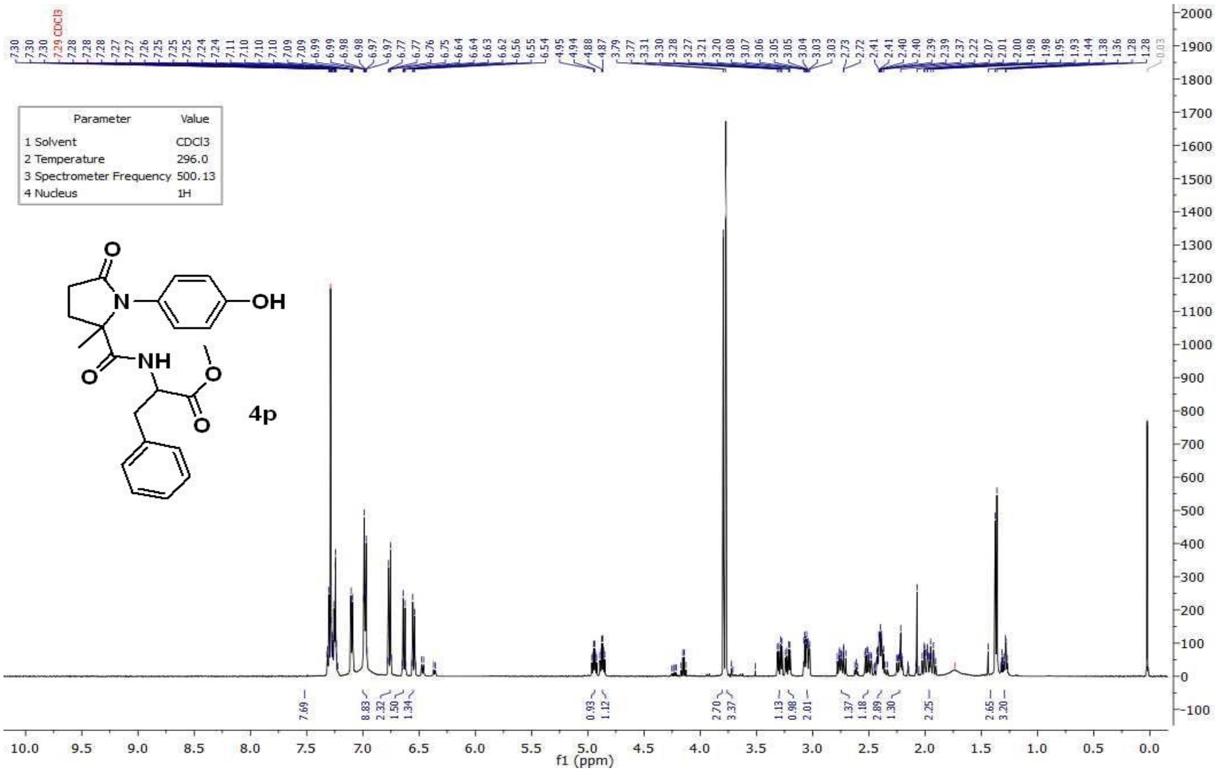

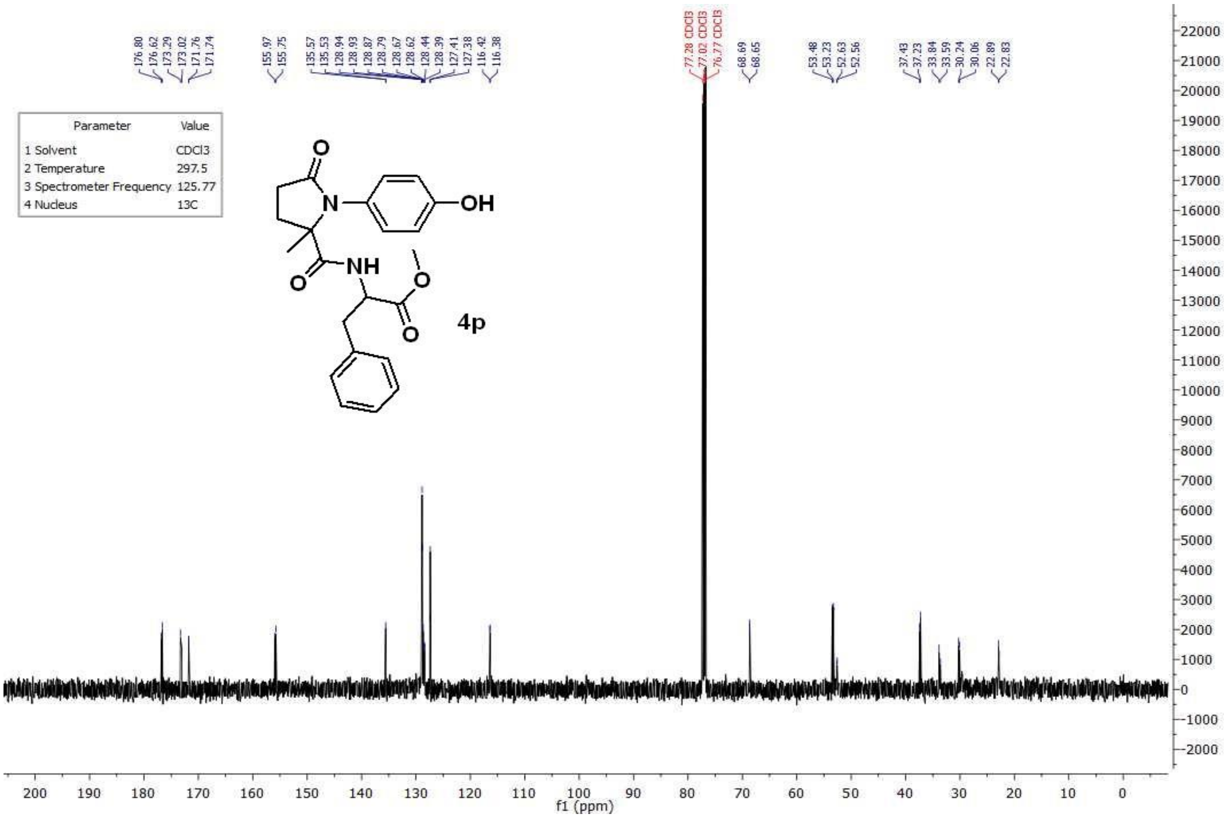

X-ray crystallography

Crystal data of compound (*R*,S**)-**4a** (CCDC- 1896942)

Empirical formula	$C_{24} H_{25} N_3 O_4$	
Formula weight	419.47	
Temperature	298 (2)	
Wavelength	0.71073	
Crystal system	triclinic	
Space group	*P* -1	
Unit cell dimensions	*a* = 9.6897(19) Å	α = 117.44(3) °
	b = 12.831(3) Å	β = 98.57(3) °
	c = 12.984(3) Å	γ = 103.49(3)°
Volume	1330.2(8)	
Z	2	
Calculated density	1.047 Mg/m^3	
Absorption coefficient	0.072 mm^{-1}	
F(000)	444	
Crystal size	0.500 x 0.400 x 0.200 mm^3	
Theta range for data collection	1.85 to 25.00 °	
Index ranges	-11<=h<=11, -15<=k<=13, 0<=l<=15	
Reflections collected	4648	
Independent reflections	4648 [R(int) = 0.0000]	
Data / restraints / parameters	4648/2/288	
Goodness-of-fit on F^2	0.936	
Final R indices [I>2σ(l)]	R1 = 0.1018, ωR2 = 0.2673	
R indices (all data)	R1 = 0.1717, ωR2 = 0.3041	
Largest diff. peak and hole	0.433 and -0.282 e×Å3	
